# Cathelicidin-mediated lipopolysaccharide signaling via intracellular TLR4 in colonic epithelial cells evokes CXCL8 production

**DOI:** 10.1080/19490976.2020.1785802

**Published:** 2020-07-13

**Authors:** Ravi Holani, Anshu Babbar, Graham A. D. Blyth, Fernando Lopes, Humberto Jijon, Derek M. McKay, Morley D. Hollenberg, Eduardo R. Cobo

**Affiliations:** a Production Animal Health, Faculty of Veterinary Medicine, University of Calgary, Calgary, Canada; b Microbiology and Infectious Diseases, Cumming School of Medicine, University of Calgary, Calgary, Canada; c Institute of Parasitology, McGill University, Montreal, Canada; d Physiology and Pharmacology, Cumming School of Medicine, University of Calgary, Calgary, Canada

**Keywords:** Colonic epithelium, cathelicidin, neutrophils, lipopolysaccharides, CXCL8, *salmonella* spp

## Abstract

We hypothesized that the antimicrobial peptide cathelicidin has a physiological role in regulating gut inflammatory homeostasis. We determined that cathelicidin synergizes with LPS to facilitate its internalization and signaling via endosomic TLR4 in colonic epithelium, evoking synthesis of the human neutrophil chemoattractant, CXCL8 (or murine homolog, CXCL1). Interaction of cathelicidin with LPS in the control of CXCL8/CXCL1 synthesis was assessed in human colon epithelial cells, murine colonoids and cathelicidin-null mice (*Camp^−/-^*). Mechanistically, human cathelicidin (LL-37), as an extracellular complex with LPS, interacted with lipid raft-associated GM1 gangliosides to internalize and activate intracellular TLR4. Two signaling pathways converged on CXCL8/CXCL1 production: (1) a p38MAPK-dependent pathway regulated by Src-EGFR kinases; and, (2) a p38MAPK-independent, NF-κB-dependent pathway, regulated by MEK1/2-MAPK. Increased cathelicidin-dependent CXCL8 secretion in the colonic mucosa activated human blood-derived neutrophils. These cathelicidin effects occurred *in vitro* at concentrations well below those needed for microbicidal function. The important immunomodulatory role of cathelicidins was evident in cathelicidin-null/*Camp^−/-^* mice, which had diminished colonic CXCL1 secretion, decreased neutrophil recruitment-activation and reduced bacterial clearance when challenged with the colitis-inducing murine pathogen, *Citrobacter rodentium*. We conclude that in addition to its known microbicidal action, cathelicidin has a unique pathogen-sensing role, facilitating LPS-mediated intestinal responses, including the production of CXCL8/CXCL1 that would contribute to an integrated tissue response to recruit neutrophils during colitis.

## Introduction

Cathelicidins are small cationic peptides produced by epithelial cells, macrophages and polymorphonuclear leukocytes.^1,2^ A single cathelicidin gene is present in humans (*cathelicidin antimicrobial peptid*e, *CAMP*), which yields a 37 amino acid peptide (leucine-leucine, LL-37) generated by extracellular cleavage of the C-terminus.^3^ The murine counterpart is cathelicidin-related-antimicrobial-peptide (CRAMP), encoded by the gene *Camp* (formerly *Cnlp*).^4^ LL-37 and CRAMP are considered homologous; they share similar structure (α-helical and net charge of +6) and antimicrobial capabilities and display interspecies (humans – mice) functions.^5,8^ Whereas cathelicidins are considered broad antibacterials against Gram-positive and Gram-negative bacteria,^5,9,11^ the relevance of such activity *in vivo* is controversial. These peptides have weak killing activity under physiological conditions, including high salt concentrations or in the presence of sugars.^12,13^ Cathelicidin’s bacteriocidal activity is inhibited by bacterial surface modifications such as *Salmonella spp* lipid A acylation.^14^

Apart from their direct bacteriocidal activities, cathelicidins were shown to modulate inflammatory responses, primarily in hematopoietic cells.^2^ Cathelicidins enhance chemokine expression (CXCL1, CXCL8, CCL2, CCL5 and CCL10) by monocytes and reduce TNF-α production by macrophages challenged with lipopolysaccharide (LPS), lipotechoic acid (LTA) or lipoarabinomannan.^5,15,17^ It has been proposed that cathelicidins recruit monocytes, neutrophils and eosinophils,^18,20^ possibly by activating chemotactic formyl peptide receptor 2 (FPR2).^19,21^ Although cathelicidins are produced by intestinal epithelial cells, potentially to ward off bacteria, a direct effect of cathelicidins on the gut epithelium and in the context of enteric infectious bacterial disease is unknown.

During infections caused by Gram-negative bacterial pathogens (e.g., *Salmonella typhimurium, Escherichia coli*), LPS, via Toll-like receptor 4 (TLR4) signaling, can provoke release of inflammatory cytokines.^22^ In a murine infectious colitis model, TLR4 signaling induced by *Citrobacter rodentium*, a natural attaching and effacing pathogen, promotes the recruitment of neutrophils into colonic tissue.^23^ Neutrophils have an early innate defense role in the colon controlling the bacterial burden,^24,25^ in part by regulating fecal shedding and dissemination of pathogens to extra-intestinal sites (mesenteric lymph nodes and liver).^26,27^ In humans, neutrophil migration is largely regulated by the interaction of chemokine CXCL8 with CXCR1/2 receptors. In mice, two functional homologs of CXCL8, CXCL1 (keratinocyte chemoattractant (KC)) and CXCL2 (macrophage inflammatory protein-2 (MIP-2)), are regarded as early-phase neutrophil chemokines.^28^ Of note, mice deficient in CXCL1 have reduced neutrophil recruitment to the colon following infection with *C. rodentium*.^29^ The unresolved conundrum is how the colonic epithelium can respond to invasion by Gram-negative enteric pathogens, while maintaining tolerance to LPS continuously released from commensal bacteria.^30,31^ In this work, we explored the hypothesis that cathelicidin produced in response to pathogens invading colonic mucosa has a protective role in the setting of *C. rodentium* induced colitis. Our postulate was prompted by the observation that cathelicidin-deficient mice (*Camp^−/-^*), have an exacerbated diarrhea when challenged with either a colitis-inducing chemical or infectious agent (e.g. *Clostridium difficile*).^32,33^ Here we examined signal transduction pathways and other mechanisms whereby cathelicidin, in concert with LPS, can upregulate the neutrophil chemokine, CXCL8/CXCL1, contributing to the influx of neutrophils during infectious colitis.

## Results

### Cathelicidins contributed to neutrophil recruitment and pathogen clearance in infectious colitis

**Figure 1.**
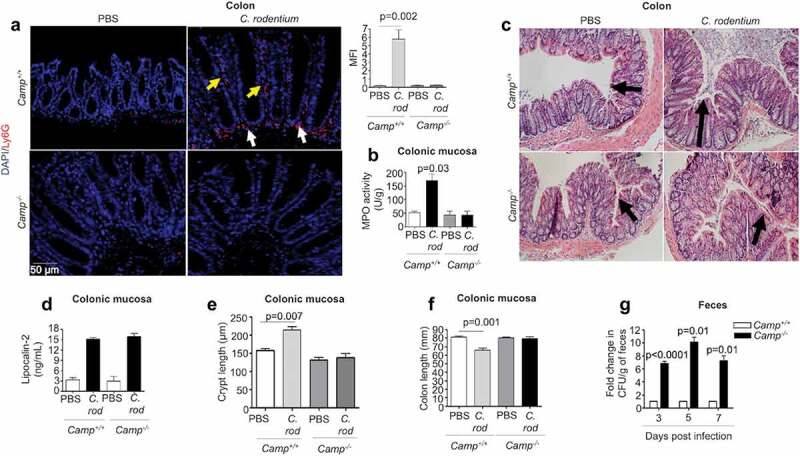
Neutrophil activation, histopathology and fecal shedding in *Camp^+/+^* and *Camp^−/-^* mice infected by *Citrobacter rodentium*. C57BL/6 *Camp*^+/+^ and *Camp^−/-^* mice were orally infected with *C. rodentium* (1 x 10^8^ CFU in 200 μL of PBS) for 7 d. (a) Immunofluorescence neutrophil staining of murine colons with anti-Ly6 G antibody (5 μg/mL). Neutrophils were widely distributed in colon (lumen, crypt and lamina propria) although this representative image only shows neutrophils along crypts (yellow arrows) and in lamina propria (white arrows). Fluorescence was calculated using ImageJ 1.50i software and represented mean fluorescence intensity (MFI). (b) MPO activity in murine colonic mucosa after 7 d pi with *C. rodentium*. Data are represented as absolute values (MPO activity (U/g)) with respect to *Camp*^+/+^ PBS control. (c) H&E microphotography of murine colons. Black arrows denote crypt length. (d-f) Histograms representing absolute values of lipocalin-2 (ng/mL) secretions in feces (d), crypt length (e) and colon length (f) in control and infected mice. (g) For *C. rodentium* burden, fresh fecal pellets (1/per mouse) were collected at 3, 5 and 7 d pi (n = 4 mice/group) and serially diluted in sterile PBS, plated on McConkey agar and counted to obtain bacterial CFU/g. Data were normalized to infected *Camp^+/+^* mice and represented as fold change in CFU/g of feces. One fold change represents ~10^5^, 10^6^ and 10^7^ CFU/g for 3, 5 and 7 d pi, respectively. Data are shown as means ± SEM (*n* = 4 mice/group). *P* < .05 (one-way ANOVA *post hoc* Bonferroni correction for multiple group comparison or two-tailed Student’s *t-*test for two groups) was considered significant.

Neutrophils are necessary for controlling *C. rodentium* in mice; for example, mice rendered neutropenic due to anti-Gr-1 antibody treatment or depletion of CXCR2 have higher colonic bacterial burdens compared to wildtype mice.^27,29^ In the present study, cathelicidin-null (*Camp^−/-^*) mice had reduced neutrophil infiltration into the distal colon compared to *Camp^+/+^* mice at peak *C. rodentium* infection (7 d post-infection; pi), as determined by immunofluorescence (Figure 1a) and myeloperoxidase (MPO) activity (Figure 1b). Further, whereas infected *Camp^+/+^* and *Camp^−/-^* mice had comparable histopathology (Figure 1c), expression of the cell damage marker lipocalin 2 (Figure 1d) and reductions in cecum weight (Fig S1B), only infected *Camp^+/+^* mice displayed increased crypt hyperplasia (Figure 1e) and decreased colon length (figure 1f) as prominent hallmarks of colitis. Bacterial burden was also affected by the absence of endogenous cathelicidins. Fecal shedding of *C. rodentium* was ~8-fold greater in *Camp^−/-^* mice compared to *Camp^+/+^* mice at 3, 5 and 7 d pi (Figure 1g). Further, *C. rodentium* was isolated from the spleen and liver of all *Camp^−/-^* mice but only in 50% of *Camp^+/+^* mice (both for spleen and liver) (Fig S1C). Lack of cathelicidins in *Camp^−/-^* mice impacted not only the number of neutrophils infiltrating the colon but also their function. Neutrophils from *Camp^+/+^* mice or a 1:1 mix of neutrophils from *Camp^+/+^* and *Camp^−/-^* mice were more effective at *in vitro* killing of *C. rodentium* compared to *Camp^−/-^* neutrophils (at 30 min) (Fig S1A). Thus, cathelicidins can contribute to the influx of neutrophils and the clearance of *C. rodentium*.

### Cathelicidins synergized with LPS to stimulate murine CXCL1 or human CXCL8 chemokines in colon

**Figure 2.**
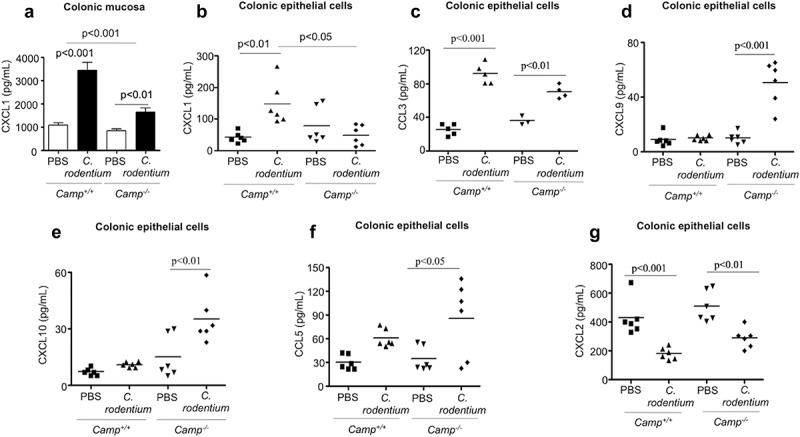
Determination of chemokine production in colonic mucosa and primary colonic epithelial cells in *Camp*^+/+^ and *Camp^−/-^* mice challenged by *Citrobacter rodentium. Camp^+/+^* and *Camp*^−/-^ mice were infected with *C. rodentium* (1 x 10^8^ CFU in 200 μL of PBS) for 7 d. (a) CXCL1 secretions in distal colonic mucosa of infected *Camp^+/+^* and *Camp^−/-^* mice (ELISA). Chemokine production of CXCL1 (b), CCL3 (c) CXCL9 (d), CXCL10 (e), CCL5 (f) and CXCL2 (g) in isolated primary CD326^+^CD45^−^ colonic epithelial cells (multiplex bead-based assay). Data are shown as mean ± SEM (*n*= 3–6/group). *P* < .05 (one-way ANOVA *post hoc* Bonferroni correction for multiple group comparisons or two-tailed Student’s *t*-test for two groups) was considered significant.

Based on *in vitro* studies, cathelicidins may influence migration of neutrophils to inflamed colons by chemoattraction, either directly via activation of FPR2 or indirectly by inducing pro-inflammatory cytokines (e.g., IL-1β, TNF-α, CXCL1).^17,19,34^ Whereas *fpr2^−/-^* and wildtype mice have similar colonic neutrophil recruitment during *C. rodentium* infection,^35^ excluding an impact of cathelicidins on FPR2 activation, the role of cathelicidins in inducing the production of chemoattractants has been less explored. In our studies, we observed increased CXCL1 secretion in colons of *Camp^+/+^* mice compared to *Camp^−/-^* mice at the peak of *C. rodentium* infection (7 d pi) (Figure 2a). Other pro inflammatory cytokines, IL-1β (Fig S1D) and TNF-α (Fig S1E) were similarly increased in the colon after infection of *Camp^+/+^* and *Camp^−/-^* mice. To determine the source of this differential expression of CXCL1, we assessed CXCL1 secretions in CD326^+^CD45^−^ epithelial cells and CD45^+^ CD326^−^ leukocytes isolated from colons of mice infected with *C. rodentium* (7 d pi). CXCL1 secretions were increased in colonic epithelial cells (Figure 2b) and leukocytes from the lamina propria (Fig S1 F) of infected mice compared to uninfected controls; however, only in colonic epithelium was the amount of CXCL1 statistically significantly higher in infected *Camp^+/+^* mice compared to *Camp^−/-^* (Figure 2b). Thus, whereas baseline constitutive *Camp* peptide expression was higher (~4-fold) in lamina propria leukocytes compared to colonic epithelium in *Camp^+/+^* mice (Fig S1 G), we decided to assess if the colonic epithelium, an early sensor of enteric pathogens (*S. typhimurium* and *E. coli*) and producer of cathelicidin,^36,38^ had a particular role in producing chemokines via the synthesis of cathelicidin.^39^ We determined that in addition to CXCL1 (Figure 2a), colonic epithelial cells from *Camp^+/+^* mice infected with *C. rodentium* produced higher amounts of the related chemokine, CCL3 (Figure 2c). In contrast, the production of CXCL9, CXCL10 and CCL5 was increased in colonic epithelium from infected *Camp^−/-^* mice ((Figure 2d-f), respectively). Kinetics of the other neutrophil chemoattractant, CXCL2, did not differ between colonic epithelium from infected *Camp^+/+^* and *Camp^−/-^* mice (Figure 2g). Thus, in addition to immune and stromal cells producing chemotactic factors and initiating inflammation (i.e. neutrophil recruitment), colonic epithelial cells particularly increase CXCL1 via cathelicidin.

**Figure 3.**
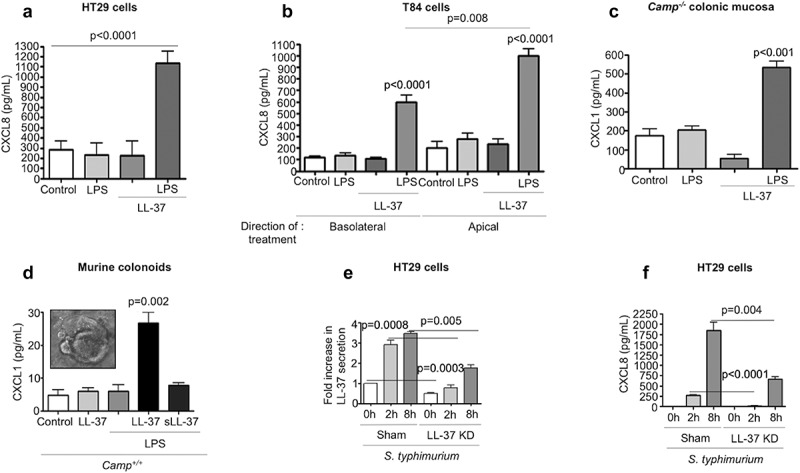
CXCL1/CXCL8 synthesis in colonic epithelium stimulated with cathelicidins and LPS or *Salmonella typhimurium* infection. (a-b) CXCL8 were determined by ELISA in cell supernatants from human colonic HT29 epithelial cells (a) and human colonic T84 epithelial cells (b). Challenge consisted of LL-37 (10 μg/mL) and LPS (1 μg/mL), either alone or in combination for 4 h. (c-d) CXCL1 protein expression determined by ELISAs in homogenized colons of *Camp^−/-^* mice (*n* = 3–5 mice/per group) (c) and colonoids from *Camp^+/+^* mice (d) treated with LPS (1 μg/g or 1 μg/mL), LL-37 (1 μg/g or 10 μg/mL) or scrambled-sequence peptide sLL-37 (10 μg/mL; only in mouse colonoids), either alone or in combination (intraperitoneally for 3 h in (C) and *ex vivo* for 4 h in (D)). (e-f) HT29 cells either transfected with sham plasmid/knocked-down for LL-37 (ShLL-37) or untransfected were challenged with *S. typhimurium* (MoI 10:1; 2 × 10^7^ CFU) for variable time points (up to 8 h). Bar graphs are representing LL-37 secretions (1 fold ~250 pg/mL) (e) and CXCL8 secretions (f) in cell supernatants. Graphs are representative of three independent experiments. Data are shown as mean ± SEM (*n* = 3 independent experiments done in triplicate, unless mentioned otherwise). *P* < .05 (one-way ANOVA *post hoc* Bonferroni correction for multiple group comparison or two-tailed Student’s *t*-test for two groups) was considered significant. ND = “not detected”.

Given the importance of TLR4 signaling and chemokine synthesis in neutrophil recruitment during enteric bacterial infections,^23,27,29^ we hypothesized that a combination of cathelicidin and LPS (LPS+LL-37) could synergize to regulate CXCL1 (murine) or CXCL8 (human). Single addition of LPS, LL-37 or a scambled LL-37 peptide, did not increase secretion of CXCL8 by human colon-derived epithelial cell lines. Remarkably, a combination of LPS+LL-37 given simultaneously increased CXCL8 secretion in 2 colonic epithelial cell lines: HT29 (early (4 h) (Figure 3a) and later (16 h) (Fig S2A)) and T84 cells (where only apical secretion was observed; Figure 3b). Synergistic increases in CXCL8 secretion were observed in HT29 cells with heat-killed *S. typhimurium* and LL-37 (Fig S2B). *E. coli* (O111:B4)-LPS also had similar synergistic synthesis of CXCL8 when combined with LL-37 (colonic HT29 cells at 4 h; data not shown).

Due to interspecies functionality and reported immunological similarities between CRAMP and LL-37,^5,19,34^ we tested whether LL-37 synergized with LPS to induce chemokines in murine colons. Intraperitoneal injection with LPS and synthetic LL-37, but neither LPS nor LL-37 alone, increased CXCL1 secretion in colons of *Camp^−/-^* mice (Figure 3c). However, neither LPS or LL-37 (alone or in combination) increased neutrophil MPO activity above baseline (PBS only control) in colons (Fig S2 C). LPS+LL-37 increased CXCL1 in colonoids developed from *Camp^+/+^* mice (4 h) (Figure 3d), whereas LPS combined with scrambled LL-37 (only (Figure 3d)) had no such effect on CXCL1 (Figure 3c-d). Synergy between Gram-negative bacteria and cathelicidins was evident in HT29 cells knocked-down (KD) in endogenous cathelicidin (ShLL-37) (Figure 3e). ShLL-37 KD cells had reduced secretion of CXCL8 when exposed to *S. typhimurium* compared to sham-transfected cells at early (2 h) and later (8 h) (Figure 3f) time points, with no cytotoxic side effects (Fig S2D).

Such synergistic CXCL8 synthesis with LPS+cathelicidins was not observed in leukocytes; in contrast, a decrease in TNF-α secretion was detected in phorbol 12-myristate-13-acetate (PMA) differentiated THP-1 cells treated with LPS+LL-37 (Fig S2E). Thus, LL-37 (and CRAMP) synergized with LPS or LPS^+^ bacteria to promote substantial production of neutrophilic chemoattractants, CXCL8 or CXCL1, from human and murine colonic epithelia, respectively.

### LPS and LL-37 through extracellular interaction induced CXCL8 secretion via TLR4

**Figure 4.**
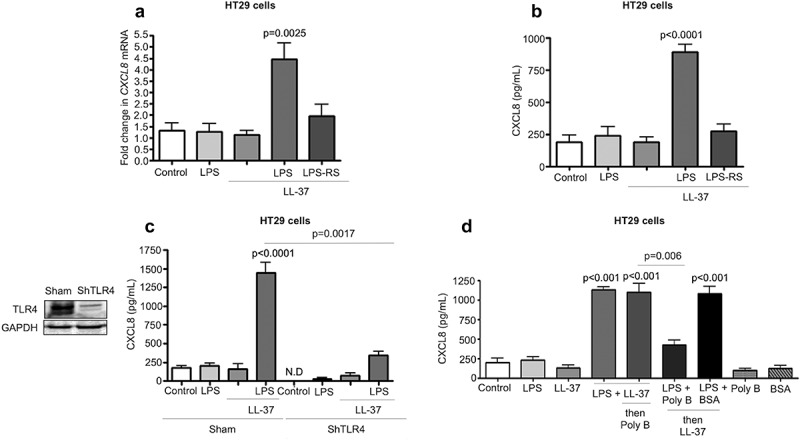
TLR4 dependent CXCL8 synthesis in colonic epithelial cells stimulated with cathelicidins and LPS. (a-d) HT29 cells used were either normal (A-B and D) or sham transfected/knocked-down in TLR4 (c). (a-c) Cells were pre-treated with LPS-RS (5 μg/mL) (or not; in C) followed by LPS (1 μg/mL) and LL-37 (10 μg/mL) alone or in combination. (d) Cells were treated by a mix of either LPS (1 μg/mL) or LL-37 (10 μg/mL) (1 h, 37°C) followed by substitution with polymyxin B (Poly B; 10 μg/mL) or LPS (1 μg/mL)+polymyxin B (Poly B; 10 μg/mL)/bovine serum albumin (BSA; 10 μg/mL) (1 h, 37°C) followed by LL-37 (10 μg/mL). Polymyxin B alone and BSA were used as negative controls. (a) *CXCL8* mRNA levels were assessed by qPCR. *GAPDH* was used as housekeeping control. (b-d) CXCL8 secretion was quantified by ELISA after 4 h. Data are shown as means ± SEM (*n* = 3 independent experiments done in triplicate). *P* < .05 (one-way ANOVA *post hoc* Bonferroni correction for multiple group comparison or two-tailed Student’s *t*-test for two groups) was considered significant.

Cathelicidins modulate colonic epithelial TLR4 in the presence of LPS, *Salmonella* spp. or *E. coli*.^40,41^ Herein, there was a key role of colonic epithelial TLR4 for cathelicidin-induced CXCL8 secretion. Inhibition of TLR4 with LPS-RS blocked *CXCL8* mRNA and protein synthesis in HT29 cells challenged with LPS+LL-37 (Figure 4a-b). Further, TLR4 KD (ShTLR4) HT29 cells had diminished CXCL8 production when stimulated with LL-37+LPS compared to sham-transfected controls (Figure 4c). The TLR4 co-adaptor protein MD-2 was not required for the production of CXCL8, as determined by a selective pharmacological MD-2 antagonist, L48H37 (Fig S3A). Secretion of the TLR4 accessory LPS-binding protein (LBP) was negligible in HT29 cells, irrespective of treatment (data below detection) and therefore could not account for the action of LPS/cathelicidin complex. When testing other TLR ligands and production of chemokines by HT29 cells, LL-37 reduced TLR9 agonist oligodinucleotide (ODN) mediated CXCL8 secretion but had no effect (synergistic or antagonistic) with the TLR5 agonist flagellin (Fig S2A). We concluded that the combined action of LPS+cathelicidin was due to TLR4 activation.

The next step was to determine if LPS bound to LL-37 to form a non-covalent complex prior to interacting with its colonic epithelium target. To this end, we first treated LPS with polymyxin B, a cyclic cationic polypeptide that binds to and neutralizes LPS. Prior treatment of LPS with polymyxin B prevented its ability to work in combination with cathelicidin to stimulate CXCL8 production by HT29 cells (Figure 4d). Conversely, if LPS and cathelicidin were first allowed to interact, followed by the addition of polymyxin B, the combined action of LPS+cathelicidin to enhance CXCL8 production in HT29 cells was not affected (Figure 4d). These data implicated the extracellular formation of a polymyxin B resistant complex between LPS and LL37 to stimulate CXCL8 synthesis.

### LL-37 promoted CXCL8 synthesis via monosialotetrahexosylganglioside (GM1)-lipid raft mediated LPS uptake and intracellular TLR4 signaling

**Figure 5.**
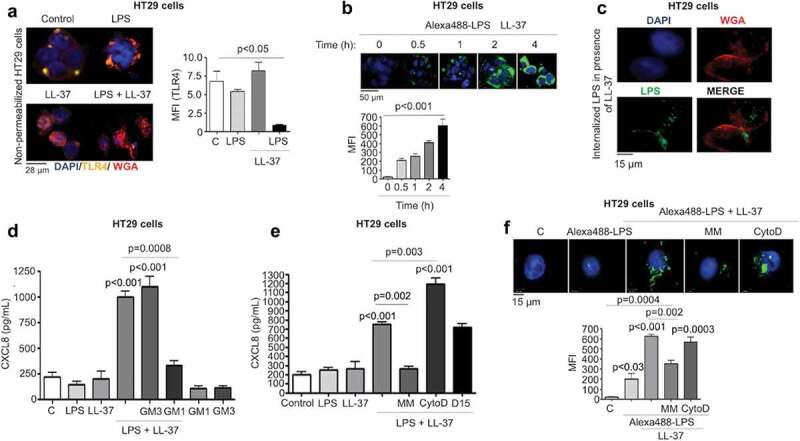
LL-37-mediated GM1-lipid raft dependent LPS uptake regulates CXCL8 synthesis via intracellular TLR4 signaling in colonic epithelial cells. (a) Fluorescent images depicting TLR4 localization in non-permeabilized HT29 cells after LPS (1 μg/mL) ± LL-37 (10 μg/mL) for 4 h (anti-TLR4 antibody; yellow). A bar graph representing quantification of TLR4 fluorescence as mean fluorescence intensity (MFI). (b-c) HT29 cells were challenged with a combination of Alexa488-conjugated-LPS (1 μg/mL) and LL-37 (10 μg/mL) for variable intervals (up to 4 h) followed by quantification of fluorescence (b) or combined with wheat germ agglutinin (WGA) as a membrane marker (for 4 h) (c). (d) CXCL8 was quantified by ELISA in supernatants from HT29 cells treated with LPS and LL-37 ± exogenous monosialotetrahexosylganglioside (GM1; 100 μg/mL). GM3 (100 μg/mL) was used as a negative control. (e-f) HT29 cells were pre-treated with a mix of lipid raft inhibitors (MM: methyl-β-cyclodextrin (500 μM) and mevinolin (250 ng/mL)), or endocytosis inhibitors cytochalasin D (CytoD; 2 μM), or dynamin-dependent endocytosis inhibitor (D15; 10 μM; only for (e)) for 1 h, followed by stimulation with nonconjugated LPS (1 μg/mL) (E) or Alexa488-conjugated-LPS (1 μg/mL) (f) and LL-37 (10 μg/mL), either alone or in combination for 4 h. (E) CXCL8 protein secretion was quantified using ELISA. (b and f) LPS uptake was assessed using immunocytochemistry and quantified using ImageJ 1.50i software. Fluorescence was calculated and presented as mean fluorescence intensity (MFI) for three independent experiments. Data are shown as means ± SEM (*n* = 3 independent experiments, done in triplicate). *P* < .05 (one-way ANOVA *post hoc* Bonferroni correction for multiple group comparisons or two-tailed Student’s *t* test for two groups) were considered statistically significant.

We inferred that LL-37 interacted with LPS prior to its activation of TLR4 to induce CXCL8 production. Therefore, we sought to determine how this complex reached TLR4, which can have an intracellular location for its action.^42,43^ We speculated that an LPS-cathelicidin complex may be internalized, as cathelicidins can act as a cell-penetrating peptide carrying cargo into cells.^44^ Additionally, the sequence of cathelicidin has a distant relationship to the cholera toxin B-subunit (CTB), known to internalize the A subunit of cholera toxin via ganglioside GM1 cell surface binding.^45,46^ We, therefore, hypothesized that LL-37+LPS complexes interact with lipid rafts and associated membrane GM1 gangliosides to internalize into epithelia and promote intracellular TLR4 signaling. For this, we determined intracellular TLR4 expression in HT29 cells was ~3-fold higher than cell surface expression (Fig S3B). Surface staining of TLR4 diminished in HT29 cells after challenge with LPS+LL-37, but not with LPS or LL-37 alone (Figure 5a). Furthermore, Alexa488-conjugated-LPS in combination with LL-37 (Figure 5b), but not on its own or together with a scrambled LL-37 (Fig S3 C), was time-dependently internalized into HT29 cells. Internalized LPS was visualized within cells at higher magnification using a membrane marker (Figure 5c). Addition of exogenous GM1 (100 μg/mL) to compete with LL-37+LPS for lipid rafts, reduced CXCL8 secretion (Figure 5d). Moreover, chemical lipid raft disruption with a mixture of methyl-β-cyclodextrin (500 μM) and mevinolin (250 ng/mL) (MM) abolished the ability of LPS+LL-37 to induce CXCL8 secretion (Figure 5e) and internalization of Alexa488-conjugated-LPS (Figure 5f).^47^ Other endocytic pathways, including dynamin GTPase-dependent or actin-dependent processes assessed by pharmacological inhibition with dynamin inhibitor (D15) or actin assembly inhibitor cytochalasin D (CytoD) respectively, were not involved in CXCL8 synthesis (Figure 5e) or uptake of Alexa488-conjugated-LPS by HT29 cells (only cytochalasin D; Figure 5f). In regards to cholera toxin, unlike LL-37, CTB did not synergize with LPS to enhance CXCL8 synthesis by HT29 cells (Fig S3D). Previous studies have shown that CTB facilitates the internalization of LPS and thus, activation of caspase-11 in murine macrophages.^48^ However, we observed no caspase-11 cleavage (activation) in primary colonic epithelium (i.e. murine *Camp^−/-^* colonoids) after LPS and LL-37 treatments, either alone or in combination (up to 2 h) (Fig S3E). In conclusion, LPS+LL-37 synergism in promoting CXCL8 production by colonic epithelial cells was dependent on extracellular binding with GM1-containing lipid rafts and intracellular TLR4 signaling.

### LL-37/LPS complex initiated p38MAPK signaling to promote CXCL8 synthesis

**Figure 6.**
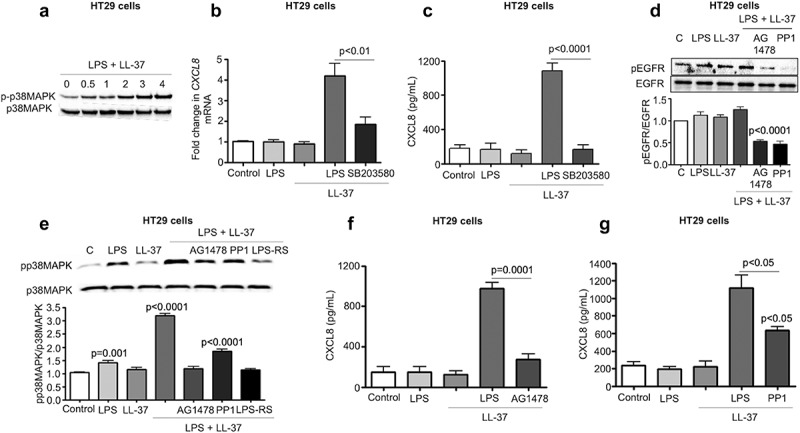
Src-EGFR kinase-mediated p38MAPK signaling promotes CXCL8 synthesis in colonic epithelial cells stimulated by cathelicidins and LPS. (a) Activation of p38MAPK in HT29 cells stimulated by LPS+LL-37 was detected by western blotting with antibodies against phosphorylated p38MAPK. Total loading was confirmed after immunoblotting with p38MAPK. (b-g) HT29 cells were pre-treated with SB203580 (10 μM) (b-c), AG1478 (1 μM) (d-f), the Src kinases inhibitor PP1 (1 μM) (D-E and G) or LPS-RS (5 μg/mL) (e) for 1 h, followed by LPS (1 μg/mL) and LL-37 (10 μg/mL) treatment. CXCL8 mRNA synthesis was quantified by qPCR after 2 h (n = 4) (b) or CXCL8 protein secretion (n = 4) by ELISA after 4 h (c). (d) Activation of EGFR was assessed after 2 h using western blotting for phosphorylated EGFR. Normalization was done with total EGFR. (E) Activation of p38MAPK was assessed after 2 h using western blotting for phosphorylated p38MAPK. p38MAPK was blotted as housekeeping control. (f-g) Total CXCL8 secretions were quantified using ELISA. Data are shown as means ± SEM (*n* = 3 independent experiments done in triplicate, unless mentioned otherwise in respective sub-figure). *P* < .05 (two-tailed Student’s *t-*test for two groups) was considered significant.

Intracellular signaling for CXCL8 synthesis in colonic epithelium involves protein tyrosine kinases and mitogen-activated protein kinases (MAPKs),^49^ although the detailed interactive pathway is elusive. Here, LPS+LL-37 resulted in a time-dependent increase in phosphorylation of p38MAPK (up to 4 h) (Figure 6a). Moreover, p38MAPK inhibition by SB203580 blocked LPS+LL-37 induced CXCL8 mRNA and protein synthesis in HT29 cells (Figure 6b-c). Next, we focussed on how TLR4 facilitates p38MAPK phosphorylation in LPS+LL-37 induced CXCL8 secretion. Of interest was the sarcoma family kinase (Src), as it is linked to TLR4 and EGFR in human mammary epithelial cells in LPS-induced lethality.^50^ There was a trend toward increased phosphorylation activation of EGFR by LPS+LL-37 that was reduced upon inhibition of EGFR (by AG1478), as well as Src (by PP1) (data shown for 2 h; Figure 6d). Further, LPS+LL-37 induced p38MAPK activation was stronger than effects promoted by either stimuli alone (data shown for 2 h; Figure 6e). Of significance, inhibitors of the EGFR (AG1478), Src (PP1) and TLR4 (LPS-RS) all reduced phosphorylation of p38MAPK (Figure 6e); furthermore, all three inhibitors attenuated CXCL8 secretion in HT29 cells stimulated by LPS+LL-37 (Figure 6f-g). Pharmacological inhibition of other membrane-based receptors that reportedly can bind LL-37, including formyl peptide receptor-like 1 (FPRL-1), P2X purinoceptor 7 (P2X7) and the EGFR2/ErbB2 that can also be cross-activated by matrix metalloproteinases (MMP),^51^ did not affect the synergistic action of LPS+LL37 to stimulate CXCL8 secretion (Fig S4A). Taken together, we inferred that synthesis of CXCL8 in colonic epithelium by LPS+LL-37 relied on the TLR4 mediated activation of p38MAPK signaling in concert with activation of EGFR and Src kinase, which appeared to be upstream of p38MAPKinase.

### MEK1/2 mediated NF-κB activation contributed to colonic CXCL8 production induced by LL-37/LPS

**Figure 7.**
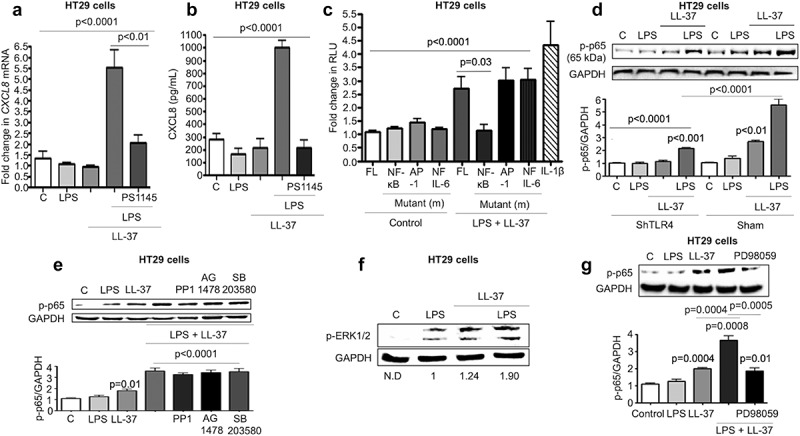
MEK1/2 kinase-dependent NF-κB activation is required for CXCL8 synthesis induced by cathelicidins and LPS in colonic epithelial cells. (a-b) HT29 cells were pre-treated with the NF-κB inhibitor PS1145 (1 μM; 1 h) followed by LPS (1 μg/mL) and LL-37 (10 μg/mL). *CXCL8* gene synthesis was quantified by qPCR after 2 h (n = 4) (a) and protein secretion by ELISA after 4 h (b). (c) HT29 cells were transfected with 174 bp wild type (FL) or mutant CXCL8 promoter construct (containing individual mutated sites for either NF-κB or AP-1 or NF-IL-6) upstream of the luciferase gene, followed by stimulation with LPS (1 μg/mL) and LL-37 (10 μg/mL) for 4 h. Data are represented as fold change in relative light units (RLU), normalized to renilla luciferase transfection control. IL-1β (10 ng/mL) was used as positive control. (d-e) HT29 cells sham transfected/knocked down for TLR4 (ShTLR4) (d) or treated with inhibitors for Src kinases (PP1; 1 μM), EGFR kinase (AG1478; 1 μM) and p38MAPK (SB203580; 2 μM) for 1 h (e) were stimulated by LPS+LL-37, either alone or in combination for 2 h. p65 phosphorylation was assessed by western blotting with specific antibodies. (f-g) HT29 cells normal and/or inhibited for ERK1/2 (PD98059; 20 μM) (only (g)) were stimulated by LPS+LL-37, either alone or in combination for 2 h. ERK1/2 (f) or p65 phosphorylation (G) was assessed by western blotting with specific antibodies. Total loading was confirmed after blotting for GAPDH. Data are shown as means ± SEM (*n* = 3 independent experiments done in triplicate). *P* < .05 (one-way ANOVA *post hoc* Bonferroni correction for multiple group comparison or two-tailed Student’s *t*-test for two groups) were considered significant.

Activation of nuclear factor kappa-light-chain-enhancer of activated B cells (NF-κB) is essential for CXCL8 synthesis in airway and cervical epithelial cells.^52^ In agreement with these findings, LPS+LL-37 induced a time-dependent phosphorylation of the p65-NF-κB subunit in HT29 cells (Fig S4B). Pharmacological inhibition of NF-κB-activating IκB kinase β (IKK) complex by PS1145 blocked the upregulation of *CXCL8* mRNA and protein secretion in HT29 cells (Figure 7a-b). We thus assessed the ability of LL-37+ LPS to stimulate NF-κB promoter elements upstream of *CXCL8* in HT29 cells using luciferase reporter constructs.^53^ Luciferase reporter activity was abolished upon mutation of the NF-κB promoter site, whereas it remained active after mutations of the activator protein (AP)-1 or nuclear factor for IL-6 expression (NFIL-6) sites (Figure 7c). This LPS+LL-37-mediated NF-κB p65 subunit phosphorylation was partially and significantly downregulated in TLR4 KD cells (Figure 7d).

Inhibition of Src, EGFR kinase and p38MAPK had no effect on LL-37+ LPS-induced phosphorylation of NF-κB/p65 (Figure 7e). In search of an alternate signal pathway(s) to regulate NF-κB, we determined LPS+LL-37 enhanced phosphorylation-activation of extracellular signal-regulated kinase 1/2 (ERK1/2), downstream of mitogen-activated protein kinase (MEK) 1/2 (Figure 7f). Moreover, pharmacological inhibition of MEK1/2 by PD98059 blocked phosphorylation-transactivation of the NF-κB subunit p65 (Figure 7g) in HT29 cells exposed to LPS+LL-37 with a concomitant reduction in CXCL8 secretion (Fig S4 C). Similarly, phosphorylation-transactivation of the NF-κB subunit p65 was observed in LPS-challenged bone marrow-derived macrophages from *Camp^+/+^*, but not *Camp^−/-^* mice (Fig S4D). The proposed signaling pathways were verified in colons of *C. rodentium*-infected mice, where increased phosphorylation-activation of EGFR (a component of p38MAPK signaling axis) and NF-κB (p65) occurred in *Camp^+/+^* compared to *Camp^−/-^* mice (Fig S4E). Thus, synergistic CXCL8 synthesis in colonic epithelium stimulated by LPS+LL37 was contingent upon two signals: p38MAPK activation (regulated by Src and EGFR kinases) and p38MAPK-independent NF-κB activation, downstream of TLR4 and MEK1/2 MAPK.

### Secreted colonic CXCL8 activated human blood-derived neutrophils

**Figure 8.**
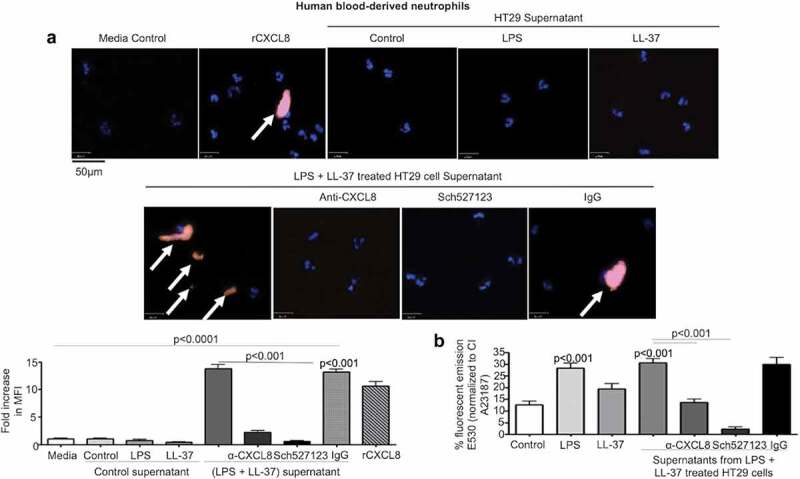
Secreted CXCL8 from colonic epithelium stimulated by cathelicidins and LPS induces calcium flux and activation of neutrophils. (a-b) Human neutrophils (1 x 10^4^/well in an 8-well chamber) were incubated at 37° C for 1 h alone (a) or pre-incubated with Fluo4-NW dye (1 x 10^6^ cells, 45 m, RT) (b). Neutrophils were exposed to supernatants from HT29 cells unstimulated (control) or stimulated with LPS (1 μg/mL) and LL-37 (10 μg/mL), either alone or in combination, for 4 h ± anti-CXCL8 antibody (1 μg/mL) or CXCR1/2 inhibitor SCH527123 (20 μM). rCXCL8 was used as positive control. IgG was used as an isotype control. (A) Neutrophil activation was assessed by neutrophil elastase secretion (depicted by white arrows) using immunocytochemistry with anti-neutrophil elastase antibody (5 μg/mL). Data are represented as fold increase in MFI normalized to respective control, for three independent experiments. (B) Data are represented as percentage fluorescence emission at 530 nm of positive control calcium ionophore A23187 (CI A23187). Data are shown as means ± SEM (*n* = 3 independent experiments done in triplicate). *P* < .05 (one-way ANOVA *post hoc* Bonferroni correction for multiple group comparison or two-tailed Student’s *t-*test for two groups) was considered significant.

CXCL8 is a potent chemokine that activates a G_q_-protein coupled CXCR1/2 receptor expressed on the surface of neutrophils.^54^ This action promotes calcium mobilization in neutrophils which kills microbial pathogens.^55,56^ To determine the biological significance of CXCL8 secreted by colonic epithelium when stimulated with LPS+LL-37, we assessed neutrophil responses. Secreted products released by HT29 cells in response to LPS+LL-37 promoted the secretion of neutrophil elastase (Figure 8a) and increased calcium flux (Figure 8b) in naïve blood-derived human neutrophils. This response was mostly attributed to CXCL8, since an anti-CXCL8 antibody and a CXCL8 receptor antagonist blocked both events (Figure 8a-b). Taken together, LPS+LL-37 enhanced colonic epithelial cell release of bioactive CXCL8 at levels capable of activating neutrophils.

## Discussion

**Figure 9.**
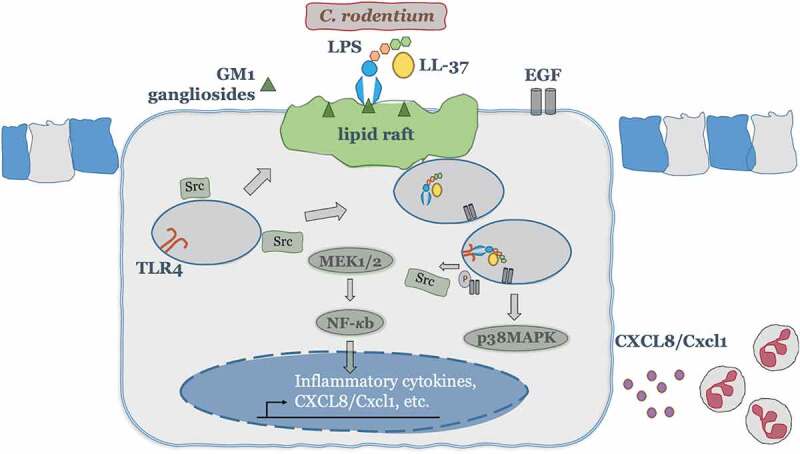
A theoretical scheme of signaling mechanisms elicited by cathelicidins in synergy with LPS to promote CXCL8 secretion in colonic epithelium and subsequent neutrophil recruitment/activation. In colonic epithelial cells, LL-37 physically binds and facilitates LPS uptake via interaction with GM1 (monosialotetrahexosylganglioside) in lipid rafts, where EGFR and Src kinases reside. Intracellularly, LPS interacts with TLR4 to promote activation of two signaling axes: one p38MAPK dependent, which relies on Src and EGFR kinases cross-talk and subsequent p38MAPK activation; second NF-κB dependent, which signals via MEK1/2 but independent of Src, EGFR and p38MAP kinases. Both signaling pathways together promote colonic CXCL8 protein synthesis and secretion. The induced colonic CXCL8 chemokine regulates intestinal defenses via neutrophil recruitment/activation.

This study demonstrates that endogenous cathelicidin/LL-37, beyond its bactericidal activities when secreted by hematopoietic and non-hematopoietic cells, synergizes with LPS by promoting LPS internalization into colonic epithelium via lipid rafts. The LPS then initiates intracellular TLR4 signaling which in turn elevates the production of neutrophil chemoattractants (human CXCL8 or murine CXCL1). This mechanism seems key in infectious colitis. Studies on cathelicidin-null (*Camp^−/-^*) mice had shown endogenous cathelicidin is needed for controlling *C. rodentium* infection, mostly attributed to antimicrobial actions.^57,58^ We confirmed that the elimination of *C. rodentium* was impaired in the absence of cathelicidin; however, we propose a hitherto unrecognized “pathogen sensing” mechanism of cathelicidin at the colonic epithelium (Figure 9). Cathelicidin from colonic epithelium in synergy with Gram-negative bacteria/LPS could initiate and/or contribute to the chemotaxis of neutrophils, either directly via known receptors (e.g., FPR2) or indirectly by releasing immune effectors (e.g., CXCL1) upon communication with local stromal or immune cells.^2,34,59^ Thus, epithelial production of cathelicidins and chemoattractants could act in alliance with other gut cells (e.g.resident or recruited leucocytes) to recruit neutrophils following infection to restrict the pathogen (e.g. *C. rodentium*) with the bystander effect of collateral inflammatory tissue damage.^60^ Indeed, we observed *Camp^+/+^* neutrophils are better at the direct killing of *C. rodentium in vitro* than *Camp^−/-^* neutrophils. In agreement, CXCL1 expression in intestinal stromal cells correlates with early neutrophil recruitment into the colonic mucosa which could migrate into the lumen to eliminate IgG opsonized virulent C. *rodentium*.^61,62^ Since leukocytes are major sources of cathelicidin, future studies should employ bone marrow chimeric mice (*Camp^+/+^* <-> *Camp^−/-^*)^63^ to determine the relative contribution of epithelial-derived verses leucocyte-derived cathelicidin and associated CXCL1 in neutrophil recruitment into the colon.

The cathelicidin-dependent synergistic increase in CXCL1 synthesis upon *C. rodentium* infection was mostly restricted to CD326^+^CD45^−^ colonic epithelium although baseline *Camp* expression was higher in CD45^+^CD326^−^ leukocytes located in the lamina propria. In fact, the effect of LPS+LL-37 inducing CXCL8 in the epithelium differed from immunosuppressive effects of LPS+LL-37 on macrophages and in a sepsis model.^2,64,66^ In this regard, TNF-α secretion was reduced following LPS+LL-37 treatment of PMA-differentiated human monocytes (THP-1), and CXCL8 synthesis was unaffected. Such disparate effects of cathelicidin in hematopoietic vs. non-hematopoietic cells could be attributed to the pleiotropic nature of cathelicidins acting via multiple cell surface (EGFR, P2X7 and FPR2) and cytoplasmic (TLR9 and GAPDH) receptors.^67^ Additionally, we observed systemic (ip.) LPS+LL-37 delivery in *Camp^−/-^* mice did not promote neutrophil recruitment/activity into the colon. Neutrophil recruitment is a muti-factorial process regulated by a localized gradient of chemokines factors, which include CXCL1 but also others such as C5a complementary protein and leukotrienes.^68^ Indeed, *Camp^−/-^* mice showed a reduced capacity to produce specific chemokines (CXCL1 and CCL3), whereas others (CXCL9 and CXCL10) were increased in *Camp^−/-^* mice, likely as a compensatory event. Thus, defective gut defense in cathelicidin-null mice was, at least in part, attributed to a reduced CXCL1-stimulated influx of neutrophils during infectious colitis and, LPS+LL-37 can drive epithelial chemokine responses at concentrations of LL-37 that were below microbicidal or cytotoxic.^69^

The synergistic effect on colonic CXCL8 synthesis by LPS/LL-37 complex seems to specifically involve signaling by TLR4. The ability of cathelicidin to promote internalization of LPS was consistent with reports that cathelicidin aggregated with DNA, possibly due to the positive charges in the cathelicidin sequence, to promote internalization of DNA into endosomal compartments wherein it triggered TLR9 signaling.^70^ However, LL-37 either reduced or did not enhance colonic CXCL8 production when elicited by oligodinucleotide (TLR9 activation) and flagellin (TLR5 ligand), respectively. Similar differential responses of cathelicidins to TLR ligands have been reported. In human bronchial epithelial cells, LL-37 increased CXCL8 secretion in the presence of the TLR1/2 ligand PAM3 CSK4,^71^ but decreased the impact of double-stranded RNAs (TLR3 ligand).^71,72^ Also, *CXCL8* mRNA synthesis was reduced by LL-37+ oligodinucelotide in colonic epithelial cells.^40^ Furthermore, in murine macrophages, LPS-containing outer membrane vesicles shed by *C. rodentium* were endocytosed, which then activated caspase-11 and mediated cell death.^73^ We did not observe caspase-11 activation and/or cytotoxicity in colonic epithelium after LPS+LL-37 treatment, suggesting LL-37 is particularly involved in signaling via endosomal TLR4 and secreting CXCL1 chemokine in colonic epithelium. It is noted that CXCL8 synthesis was still partially evident in TLR4 knockdown HT29 cells which may reflect residual TLR4 expression in knockdown cells and/or involvement of other pattern-recognition-receptors (PRR). However, future studies should be pursued in TLR4 and other PRR deficient mice to fully decipher the roles of TLRs in LPS and LL-37 synergism.

Both LPS trafficking and intracellular availability of LPS+LL-37 were crucial to enhance TLR4 signaling for CXCL8 secretion as confirmed in intestinal epithelial cells with different functional characteristics (HT29, T84), wildtype and *Camp*^−/-^ mice, and *ex vivo* murine colonoids. The surface and intracellular compartments of colonic epithelium express TLR4 but intracellular TLR4 was key for LPS+LL-37-driven chemokine synthesis.^42^ This intracellular TLR4 preponderance in the intestinal epithelium could be strategic in avoiding unwarranted inflammation.^74^ Co-factors of TLR4, such as MD-2 and LBP, were not involved in LPS+LL-37 induced CXCL8 secretion. Expression of MD-2 was negligible in intestinal epithelium and only overexpression of MD-2 in HT29 cells promoted CXCL8 secretion upon LPS challenge.^75,77^ Similarly, exogenous LBP failed to induce CXCL8 secretion in methotrexate differentiated HT29 cells challenged with LPS.^78^ Increased CXCL8 secretion did not occur in the presence of polymyxin B and LPS, perhaps due to the anti-endotoxin activity of polymyxin B. Moreover, cathelicidin or LPS acting on their own did not enhance colonic CXCL8; for instance, synthesis of CXCL8 in colonic epithelium (either mRNA transcription or protein translation) via phosphorylation of NF-κB (p65) did not occur with the addition of LPS alone (for up to 4 h). In agreement, only high concentrations of LPS (1 μg/mL for 18 h) increased CXCL8 production in previous studies with HT29 cells.^79^ Further, ip. administration of LPS did not affect colonic CXCL1 synthesis, whereas LL-37 alone reduced CXCL1 levels in *Camp*^−/-^ mice. It is likely that LPS does not increase CXCL1 in *Camp*^−/-^ mice due to the absence of cathelicidin that is required to act together with LPS. In the case of LL-37 acting alone, perhaps lower concentrations of the peptide (<20 μg/mL) may induce expression of Toll interacting-protein (Tollip, a negative regulator of TLRs), thereby reducing basal CXCL1 synthesis (unpublished data). This hypothesis remains to be tested. Therefore, LPS+LL-37 physically interacted with TLR4 to promote colonic CXCL8 secretion, independent of MD2 and LBP adaptors. This multi-checkpoint strategy in the gut might prevent unnecessary inflammation in the presence of commensals and activate the colonic epithelium (CXCL8 synthesis) only during an infectious challenge, when a Gram-negative pathogen (or its LPS) is available and can form complexes with cathelicidins.

Mechanistically, we inferred a physical extracellular interaction between LL-37 and LPS, followed by internalization of an LPS+LL-37 complex via GM1-containing lipid rafts. Precise sites of interaction between LPS and LL-37, as for the interaction between cathelicidin and self-DNA,^70^ remain to be determined. The interaction between LL-37 and other negatively charged ligands like LPS and DNA may well involve multiple lysine (K)/arginine (R) residues in LL-37. Positive charges on cathelicidins in proximity to negatively charged LPS would enable embedding of LL-37 hydrophobic helices into LPS micelles.^80,81^ Of the 10 positive lysine/arginine side chains in LL-37, three are situated immediately upstream of a negatively charged aspartic or glutamic acid, whereas four are present as dibasic clusters (FFR^140^K^141^ S; FK^151^R^152^IVQRI). The two C-terminal LL-37 basic residues are spaced by four amino acids (LR^162^NLVPR^167^TES), one of which is a proline that would generate a ‘twist’ between the two arginines, thereby limiting ligand interaction. Thus, if the positive side-chains in LL-37 were involved in an LPS or DNA interaction, the most likely sequences would be FFR^140^K^141^ S and FK^151^R^152^IVQRI. Of note, both of these sequences, but not the C-terminal arginines of LL-37 (LR^162^ NLVPR^167^TES), would also be present in the cathelicidin-derived inflammatory peptide, FA-29.^82^ In principle, both LL-37 and FA-29 could synergize with LPS to drive CXCL8/cytokine synthesis via TLR4. Our continuing work is aimed at testing these hypotheses to identify the precise site whereby LPS interacts with LL-37 and to determine if FA-29, like cathelicidin, can synergize with LPS to drive cytokine production. Based on our data, the LPS+LL-37 complex gained access to intracellular TLR4 via a GM1/lipid raft mechanism. In support of this finding, lipid rafts were necessary for LPS signaling via intracellular TLR4 in murine small intestinal crypt (m-IC_cl2_) cells and lipid rafts were involved in ability of LL-37 to promote endocytosis of LPS in lung epithelial cells.^47,83^ Further work identifying the nature of the LL-37/LPS interaction should clarify how this complex interacts with lipid raft-containing ganglioside GM1. Interestingly, ganglioside GM1-mediated lipid rafts are key in the internalization of cholera toxin A/B. However, whereas cholera toxin promoted caspase-11 activation in murine macrophages by increasing levels of LPS in the cytoplasm,^48^ LL-37 likely increases endosomal LPS trafficking in colonic epithelium as showed by insufficient caspase-11 activation. Indeed, cholera toxin and LPS did not synergistically enhance CXCL8 synthesis in colonic epithelium perhaps, due to inefficient activation of endosomal TLR4 as compared to the LPS+LL-37 complex.

Two signal pathways converged on the regulation of CXCL8 production by LPS+LL-37 in the colonic epithelium (Figure 9). First, LPS+LL-37 promoted CXCL8 synthesis via p38MAPK activation, dependent on Src and EGF receptor-kinase activity. Interestingly, p38MAPK extended the CXCL8 mRNA half-life in HT29 cells stimulated with TNF-α,^84^ whereas EGFR kinase stabilized amphiregulin mRNA in keratinocytes upon exposure to ultraviolet B radiation.^85^ Thus, our reported Src-EGFR-p38MAPK signaling could be a cathelicidin downstream effect in the regulation of colonic CXCL8 synthesis, aiding in mRNA stabilization. Second, CXCL8 synthesis was dependent on MEK1/2 and NF-κB (p65) phosphorylation/activation. Likewise, LL-37 promoted NF-κB dependent CXCL8 secretion in human monocytes.^16^ Further, we determined *Camp^+/+^* BMMs had higher NF-κB activation than *Camp^−/-^* BMMs upon LPS challenge. This contrasted with previous studies where exogenous LL-37 inhibited LPS-induced nuclear translocation of NF-κB in leukocytes.^65^ It is recognized that endogenous and exogenous cathelicidins could signal differentially on various cell types. Exogenous CRAMP diminished LPS-induced NF-κB activation in macrophages, whereas endogenous CRAMP promoted NF-κB activation.^86^ The outcome of cathelicidin-bacterial ligand(s) interaction appeared to be dependent upon the nature of the bacterial molecule, microenvironmental conditions, the cell and the receptor (sub)types it expressed.

In summary, we describe cathelicidin-mediated “pathogen sensing” in the colonic epithelium via ganglioside GM1/lipid rafts mediated LPS internalization and sequential cross-talk with intracellular TLR4. Such host–pathogen interaction at the colonic mucosa resulted in the production of the neutrophil chemoattractant CXCL8 (humans)/CXCL1 (mice) and pathogen clearance, representing a novel endogenous innate cathelicidin defense in the setting of infectious colitis.

## Methods

### Ethics statement

All studies in mice were conducted following regulations specified by the Canadian Guidelines for Animal Welfare (CGAW) and were approved by the University of Calgary Health Sciences Animal Care Committee (AC16-0092).

### Salmonella typhimurium

*S. typhimurium* (clinical strain LT2/ATCC 700720), provided by Dr. J. De Buck Univ. Calgary, was grown in Luria-Bertani (LB) Miller broth (IBI Scientific) (18 h, 37°C at 225 rpm) and streaked out on an LB agar plate.

### Murine models and tissue processing for cytokine analysis

Male 8-wk-old wild-type *Camp^+/+^* and cathelicidin-null *Camp^−/-^* C57BL/6 mice (B6.129X1-*Camp^tm^^1^^Rlg^*/J; The Jackson Laboratory) were littermates and were co-housed for several generations in a pathogen-free environment (Univ. Calgary). For systemic LPS challenge, *Camp^−/-^* mice were injected *i.p*. either with phosphate-buffered saline (PBS, Gibco, Life Technologies; 1x) as vehicle or with LPS derived from *S. typhimurium* (L6143; Sigma-Aldrich) and synthetic LL-37 amide trifluoroacetate salt peptide (>98.6% purity, H-6224.0005; Bachem) administered either alone or in combination (both at 1 μg/g). Mice were humanely euthanized 3 h post-challenge to collect colon samples. Distal colon was sampled, weighed, and suspended in either hexadecyltrimethylammonium bromide (HTAB) buffer (50 mg tissue wet-weight/mL) for myeloperoxidase (MPO) activity or, in sterile-PBS containing protease inhibitor cocktail (50 mg tissue wet-weight/mL) for ELISAs.^87^ After tissue homogenization, IL-1β, TNF-α and CXCL1 cytokines were quantified by quantitative ELISA (DY401, DY410 and DY453, respectively; R&D Systems). MPO activity was determined as described and is represented as units (U)/g.^88^ Data are presented as absolute values normalized to tissue wet weight.

For the infectious colitis model, *C. rodentium* (DBS-100) was cultured on McConkey agar plates (16 h, 37°C) and single colonies sub-cultured in LB broth (5 mL, 16 h, 37°C) without shaking. Mice were gavaged orally with 200 μL PBS (1x) or *C. rodentium* (~1 × 10^8^ CFU in 200 μL), a reported dose for establishing infection in C57BL/6 mice,^89,90^ and humanely euthanized 7 d pi. Distal colon was processed for MPO activity determination and ELISA as discussed above or in denaturing cell extraction buffer (DCEB; FNN0091; Thermo Fischer Scientific) containing protease inhibitor cocktail (50 mg/mL) for western blotting. MPO activity was determined and represented as units (U)/g.^87^ For CXCL1 and lipocalin-2 (DY1857; R&D Systems), ELISA was performed as discussed above.

Chemokine content in acutely isolated intact colonic epithelial cells was assessed by multiplex bead-based assay (Chemokine Discovery MD31, Eve Technologies). Colonic epithelial cells were recovered as described.^91^ Briefly, entire colons were aseptically removed from PBS control and *C. rodentium* infected *Camp^+/+^* and *Camp^−/-^* mice and rinsed with cold PBS (1X). Colons were cut-open longitudinally, sectioned (2-cm pieces) and suspended into isolation buffer containing EDTA (5 mM; 15575020, ThermoFisher Scientific) and DTT (0.324 M; D0632, Sigma-Aldrich) in 10 mL PBS. Isolated cells in suspension were incubated (37°C, 20 min on a rocker-shaker), passed through a cell strainer (70 μm; 10199656, VWR), washed with cold PBS (3X) and processed for downstream applications. For isolation of lamina propria cells, colon pieces devoid of epithelial cells were transferred into RPMI media, minced into (~5 mm) pieces and incubated with Liberase (100 units of activity/mL; containing collagenases I and II and Thermolysin; 5401119001; Sigma-Aldrich) (37°C in rocker-shaker, 1 h). The Liberase digestion process was repeated twice and lamina propria cells containing supernatant were collected in-between digestions. These cells were washed with cold PBS and processed for downstream applications. Purity of primary colonic epithelial and lamina propria cell populations was determined as CD326^+^ (EpCAM^+^; 563477, BDbioscience) CD45^−^ (103139, BDbioscience) and CD326^−^ CD45^+^ cells, respectively, using flow cytometry (~85–90%; data not shown). Data for multiplex chemokine assay were normalized to the total amount of protein (pg/mL).

To measure CFU/g of *C. rodentium* in tissue samples of liver and spleen, these organs were homogenized in PBS (50 mg/mL). Lysates were then plated on MacConkey agar plates at various dilutions and *C. rodentium* colonies quantified. To determine fecal shedding of *C. rodentium*, fresh fecal pellets (1 per mouse) were obtained aseptically from infected mice at 3, 5 and 7 d pi, and cultured on McConkey agar plates at various dilutions. Bacterial colonies were counted and data were represented as fold increase in CFU/g normalized to *Camp^+/+^* infected mice. A qPCR validation was done using *C. rodentium* specific primers against *espB* (extracellularly secreted protein B) gene (forward primer: 5′-ATGCCGCAGATGAGACAGTTG-3′ and reverse primer: 5′-CGTCAGCAGCCTTTTCAGCTA-3′) (data not shown).^92^

### Murine colonoids

Mini 3D-gut colonoids were developed from murine colonic crypts as described.^93^ Upon isolation, crypts were cultured on matrigel matrix for 10 d until colonoid morphology was apparent. After treatment(s), cell lysates were collected in DCEB containing protease inhibitor cocktail for western blotting, and supernatants were collected for quantification of CXCL1 by ELISAs.

### Isolation of murine bone marrow macrophages

For isolation and culture of bone marrow macrophages (BMMs),^94^ femoral and tibial bones were aseptically removed from *Camp^+/+^* and *Camp^−/-^* mice. Upon isolation, bone marrow monocytes were cultured (6 d) in Roswell Park Memorial Institute 1640 medium (RPMI; Invitrogen Life Technologies) supplemented with 10% fetal bovine serum (FBS; Benchmark Gemini Bio-Products), 2 mM l-glutamine, 50 μM 2-mercaptoethanol, 10 mM HEPES buffer (pH 7.4), 1% penicillin (100 U ml^−1^)/streptomycin (100 μg ml^−1^; HyClone Thermo, Fisher Scientific) and 10% conditioned media from L929 cells (as a source of macrophage colony-stimulating factor required for macrophage lineage differentiation). For experiments, BMMs cultured in RPMI without FBS and antibiotics were challenged with LPS (1 μg/mL) for 1 h (for immunoblotting). Cell lysate proteins were isolated using DCEB buffer and assessed for phospho-NF-κB p65-Ser536 (3033; Cell Signaling Technology) activation. GAPDH was used as housekeeping control.

### Isolation of neutrophils from murine bone marrows

Bone marrows from *Camp^+/+^* and *Camp^−/-^* mice were isolated as described above. In brief, bone marrows were laid on the top of a three-layered Percoll gradient (i.e., 72%, 64% and 52%) (17089102; Amersham Bioscience) and centrifuged (650 *x g*, 4°C, 30 min, zero de-acceleration). Neutrophils were collected from the layer between 72% and 64% Percoll, washed with PBS (1X) and resuspended in RPMI media at a concentration of 1 × 10^7^ cells/mL.

### In vitro *killing assay of* Citrobacter rodentium *by murine neutrophils*

Murine bone marrow neutrophils from *Camp^+/+^* and *Camp^−/-^* mice were isolated and purified using Percoll gradient as discussed above. Neutrophils were resuspended as individual genotypes (*Camp^+/+^* or *Camp^−/-^*) as well as in a 1:1 mixture (50% *Camp^+/+^* neutrophils and 50% *Camp^−/-^* neutrophils) in RPMI media without serum and antibiotic. Neutrophils (1 x 10^5^ cells/mL) were incubated with bioluminescent *C. rodentium* (DBS100; MoI 1; 1 × 10^5^ CFU) (30 min at 37°C), followed by assessment of *C. rodentium* luminescence relative to growth in LB media. *C. rodentium* growth in RPMI media was used as control. Data were represented as “% *C. rodentium* killing”’.

### Cell culture

The human colon adenocarcinoma-derived HT29 and T84 epithelial cell lines, and THP-1 human monocytic cells (provided by Dr. K. Chadee, Univ. of Calgary) were cultured in Dulbecco’s Modified Eagle’s Media (DMEM; Gibson, Life Technologies) and RPMI-1640, respectively, supplemented with 10% fetal bovine serum (FBS; Benchmark Gemini Bio-Products), 1% penicillin (100 U ml^−1^)/streptomycin (100 μg ml^−1^; HyClone Thermo, Fisher Scientific) in a humidified environment with 5% CO_2_. For experiments, cells were cultured either in DMEM (for colonic cells) or RPMI (for THP-1) without FBS or antibiotics.

HT29 cells were stimulated with *S. typhimurium*, at a multiplicity of infection (MoI) of 10:1 (~2 × 10^7^ CFU; OD_600_ (1) = ~0.8 × 10^8^ CFU/mL) for variable intervals, as indicated in the figures, heat-inactivated *S. typhimurium* (2 x 10^8^ CFU equivalent; 15 mins at 70°C), LPS from *S. typhimurium* (L6143, Sigma-Aldrich), LPS from *E.coli* O111:B4 (437627, Sigma-Aldrich), Alexa Fluor® 488-conjugated LPS from *S. minnesota* (L-23356; Molecular Probes), flagellin (SRP8029; Sigma-Aldrich), ODN (tlrl-2395; Invivogen) or CTB (C9903; Sigma-Aldrich). Concentrations of LL-37 (10 μg/mL) and LPS (1 μg/mL) (Fig S5A-D) and the time-point (4 h) examined (Fig S5E) were based on our previous studies of CXCL8 kinetics. Cytotoxicity induced by *S. typhimurium* (~2 × 10^7^ CFU) was verified with a Pierce^TM^ LDH Cytotoxicity Assay Kit (88953; ThermoFisher Scientific). Effects of LPS on colonic cells were further studied using a TLR4-competitive inhibitor of LPS isolated from *Rhodobacter sphaeroides* (LPS-RS, tlrl-prslps; Invivogen) and polymyxin B sulfate salt (1405–20-5; Sigma-Aldrich). These treatments were combined with synthetic LL-37 or LL-37 scrambled peptide (63708; Ana Spec). Absence of cytotoxic effects of LPS (10 ng/mL to 5 μg/mL) and LL-37 (1 to 30 μg/mL) was confirmed using a Pierce^TM^ LDH Cytotoxicity Assay Kit.

THP-1 human monocytic cells were differentiated with PMA (P8139; Sigma-Aldrich, 20 ng/mL) for 3 d followed by simultaneous treatment with LPS (1 μg/mL) and LL-37 (10 μg/mL),^95^ either alone or in combination, for 4 h. Cell supernatants were then collected and CXCL8 (DY208; R&D Systems) and TNF-α (DY210; R&D Systems) cytokine secretions were quantified as absolute values (pg/mL) using ELISA.

### Signal transduction pathways

Intracellular signaling was investigated by pharmacologically blocking human EGFR 2/ErbB2 (TAK 165, 366017–09-6; Tocris Bioscience), FPRL1 (WRW4, 2262; Tocris Bioscience), MMP (GM6001, 2983; Tocris Bioscience), P2X7 (A740003, 3701; Tocris Bioscience), EGFR-kinase (AG1478 hydrochloride, 1276; Tocris Bioscience), Src kinase (PP1, Calbiochem), MEK1/2 (PD98059, 9900; Cell Signaling Technology and U0126, 1144; Tocris Bioscience), p38 MAPK (SB203580, 1202; Tocris Bioscience), MD-2 (L48H37, SML1443; Sigma-Aldrich) and NF-κB activating kinase, i.e. IKKβ (PS-1145; Cayman Chemical). Endocytic processes were investigated by inhibiting endocytosis (D15, 2334; Tocris Bioscience), actin polymerization (Cytochalasin D, C8273; Sigma-Aldrich) and lipid raft formation (mevinolin, M2147, HMG-CoA reductase inhibitor; Sigma-Aldrich and methyl-β-cyclodextrin, 332615, cholesterol solubilizing agent; Sigma-Aldrich). Cells were pre-treated (or not) for 1 h with inhibitors at concentrations either based on their IC_50_ values as recommended by manufacturer(s) or obtained from previous studies and maintained in culture medium without FBS or antibiotics.^40,47,96^

Activation/phosphorylation of signal components (p38MAPK, ERK1/2 and NF-κB) was monitored by western blot analysis. Total proteins from cell lysates were isolated using DCEB buffer with a protease inhibitor cocktail. Extracted proteins were blotted using specific primary antibodies to detect phospho-p38 MAPK-Thr180/Tyr182 (4511; Cell Signaling Technology), p38 MAPK (9212; Cell Signaling Technology), phospho-ERK1/2 (4370; Cell Signaling Technology), phospho-NF-κB p65-Ser536, anti-caspase-11 (ab22684; Abcam) and human GAPDH-6C5 (1001; Calbiochem). Horseradish-peroxidase-conjugate (HRP) goat anti-mouse IgG (H + L) (115–035-146; Jackson ImmunoResearch) or HRP goat anti-rabbit IgG (H + L) (115–035-144; Jackson ImmunoResearch) was used as secondary antibodies and developed using the Clarity Western ECL Detection System (BioRad). Image capture and densitometric analyses were performed with ChemiDoc MP Imaging system and ImageLab 4.0.1 software (BioRad), respectively. Normalization was done with reference to either GAPDH (housekeeping protein) or respective total protein (p38MAPK). Results were reported as mean fold change of target expression in stimulated groups, compared to unstimulated control group.

### *Detection of CXCL8 protein secretion and gene transcription of* CXCL8 *and* Camp

Secretion of human CXCL8 from epithelial cells was quantified using ELISA (DY208; R&D Systems). Transcription of human *CXCL8* and murine *Camp* mRNA was quantified by quantitative real-time polymerase chain reaction (qPCR) using pre-designed primers (RT^2^ qPCR Primer Assay, Qiagen) specific for human *CXCL8* (PPH00568A; NM_000584.3), murine *Camp* (PPM25023A; NM_009921), human *GAPDH* (PPH00150 F; NM_002046.5) and murine *GAPDH* (PPM02946E; NM_008084) with verified specificity and efficiency (>95%) to ensure amplification of a single product of the correct size, as indicated in MIQE guidelines.^97^ Target gene mRNA values were corrected relative to the normalizer, *GAPDH*. Data were analyzed using the 2^−ΔΔCT^ method and reported as mean fold change of target transcript levels in stimulated groups versus untreated control group or CD45^+^ CD326^−^ leukocytes vs. CD326^+^CD45^−^ colonic epithelium for murine *Camp*.

### LPS uptake in colonic epithelium

HT29 cells were treated with Alexa488-conjugated-LPS ± LL-37 or scrambled LL-37 peptides, fixed with 4% PFA in the dark (15 min, RT), and counterstained for nuclei with 4ʹ, 6-diamidino-2- phenylindole (DAPI, 62247; ThermoFisher Scientific) (1:1,000, 30 min, RT). Slides were then examined using a wide-field immunofluorescence microscope (IX71Olympus). Assessment of Alexa488-conjugated-LPS uptake by colonic cells was performed using ImageJ 1.50i software (© National Institute of Health). Fluorescence intensity was calculated in randomly selected fields of view (five/replicate), either with a cluster of cells (for time-dependent LPS uptake assay) or single cells (for LPS uptake in presence of inhibitors) per replicate, for a total of three replicates/experiment. Data were reported as MFI for three independent experiments.

### TLR4 localization in colonic epithelium

HT29 cells (treated with LPS ± LL-37 or not) were either permeabilized (for intracellular TLR4) or not (surface TLR4), followed by analysis using immunofluorescence or flow cytometry. For immunofluorescence, cells were fixed as discussed above and permeabilized (or not) using 0.1% Triton X-100 (T8787; Sigma-Aldrich). Cells were then incubated with anti-TLR4 primary antibody (ab89455; Abcam) (1:100, O/N, 4°C) followed by cyanine 3-conjugated (Cy3) donkey anti-mouse IgG (H + L) (715–165-150; Jackson ImmunoResearch) secondary antibody (1:200, 1 h, RT). Cells were counterstained for nuclei with DAPI (1:1000, 30 min, RT) and/or Alexa647-conjugated wheat germ agglutinin (WGA; W32466; ThermoFisher Scientific) (1:500, 30 min, RT), which binds membrane expressing N-acetyl-D-glucosamine and sialic acid. Slides were examined using a wide-field immunofluorescence microscope (IX71Olympus). Data were reported as MFI for three independent experiments. For fluorescence-activated cell sorting (FACS) analysis, HT29 cells permeabilized (or not) were incubated either with PE-labeled anti-TLR4-CD284 (12–9917-41; eBioscience) or PE-labeled isotype mouse IgG (12–4724-41; eBioscience) (0.5 μg/mL, 30 min, 4°C). Cells were then analyzed using FACS (BD^TM^ LSR II; BD-Bioscience). Data were obtained as mean fluorescence intensity (MFI) and represented as TLR4 expression normalized to surface expression.

### TLR4 and LL-37 knock-down in colonic epithelial cells

A short hairpin (Sh)-TLR4 or (Sh)-LL-37 pGFP-V-RS plasmid vector or non-effective scrambled shRNA construct (sham) (TG320555 and TG314213; Origene) was transfected into confluent HT29 cells using Cell Line Nucleofector® Kit V (VCA-1003; Lonza). Transfected cells were selected for puromycin resistance (30 μg/mL). Knockdown efficiency was ~80-90 and 70–80% for TLR4 and LL-37, respectively, as assessed either through relative intensity quantification of TLR4 protein bands (ab13867; Abcam) or LL-37 ELISA (HK321; Hycult Biotechnology). Cells were maintained by constantly culturing them in DMEM media supplemented with puromycin (15 μg/mL).

### Luciferase CXCL8 promoter transfection in colonic epithelium

A pGL3 basic plasmid containing 174 bp CXCL8 promoter construct (−166 to +8 relative to transcriptional start site) upstream of a firefly luciferase gene and a pRL null plasmid constitutively expressing renilla luciferase gene were utilized (provided by Dr. D. Proud, Univ. Calgary). The CXCL8 promoter construct contained either intact (174 bp full length; FL) or individually mutated sites for NF-κB, activator protein (AP)-1 and nuclear factor for IL-6 expression (NFIL-6) (site-directed mutagenesis; denoted as mNF-κB, mAP-1 and mNFIL-6, respectively). HT29 cells were co-transfected with the construct (1 μg) and pRL (0.1 μg) in DMEM media (FBS and antibiotic free) using TransIT transfection reagent (MIR5405; Mirus Bio LLC). pRL null plasmid was used as transfection control. Recombinant human interleukin-1β (IL-1β; 10 ng/mL, 8900; Cell Signaling Technology) was used as a positive control for CXCL8 promoter assay. Results were quantified using a dual-luciferase reporter assay (PR-E1910; Promega). Data were obtained as fold change in relative light units (RLU) of firefly luciferase activity with respect to control group, for three independent experiments.

### Histological assessment and immunofluorescence in murine colon

Colonic tissues fixed in 10% neutral-buffered formalin and embedded in paraffin were stained with hematoxylin-eosin (H&E). For immunofluorescence, sections were deparaffinized, blocked in PBS-Tw containing 10% donkey serum (017–000-021; Jackson ImmunoResearch), 1% bovine serum albumin (BSA, 9048–46-8; Amresco), and 0.3 M glycine (1 h, RT), and incubated with primary Alexa647 tagged-anti-lymphocyte antigen 6 complex locus G6D (Ly6 G) antibody (5 μg/mL; MAB91671; R&D Systems) diluted in PBS (16 h, 4ºC). Slides were counterstained with DAPI and examined using IX71Olympus microscope. Integrated fluorescence intensity per mouse was calculated using ImageJ 1.50i software in five randomly selected fields of view and data were reported as MFI from n = 4 mice.

### Neutrophil activation

Human neutrophils from healthy donors were isolated using Lympholyte®-poly solution (CL5070; Cedarlane) and stimulated with supernatants (500 μL) harvested from HT29 cells, either untreated (control; serum-free DMEM media) or treated with LPS ± LL-37 (for 4 h). Immunofluorescence analysis was performed as described above, using an anti-neutrophil elastase antibody (5 μg/mL; MAB91671; R&D Systems) and secondary Alexa549-conjugated donkey anti-mouse IgG antibody. For neutrophil elastase secretion quantification, fluorescence intensity was calculated using ImageJ 1.50i software. Average fluorescence intensity per replicate was quantified in five randomly selected fields of view and represented as fold increase in MFI.

### Calcium flux assay

Neutrophils were isolated as discussed above. For calcium flux assay, neutrophils were incubated with Fluo4-no wash calcium dye (Fluo4 NW, F1242; Invitrogen) (1 mL; 45 m, RT). Then, neutrophils were washed with PBS and resuspended in calcium-magnesium-containing HBSS. Neutrophils (1 x 10^6^) were transferred into a cuvette and stimulated with supernatants (100 μL) harvested from either untreated (control; serum-free DMEM media) or LPS and/or LL-37 treated colonic epithelial HT-29 cells. To confirm the role of CXCL8, either supernatants from LPS-LL37 treated HT29 cells were blocked (or not) with anti-CXCL8 antibody (1 μg/mL, MAB208; R&D Systems) or mouse IgG1 isotype control (1 μg/mL, 5415 S; Cell Signaling Technology) (4°C; 1 h) or with CXC receptor1/2 (CXCR1/2) inhibitor SCH 527123 (20 μM, A3802; APExBIO), prior to stimulation of neutrophils. Recombinant human CXCL8 (200 ng/mL, 208-IL-010; R&D Systems) was used as a positive control. For calcium flux assay, calcium signals were monitored at an excitation wavelength of 480 nm and an emission wavelength of 530 nm, recorded with the Aminco Bowan series II fluorimeter and the AB2 software (Thermo Fisher Scientific). Data were expressed as a percentage of the emission fluorescence normalized to maximum fluorescence caused by 2.5 μM calcium ionophore A23187. All data recorded were within the range of fluorescence obtained with calcium ionophore A23187.

### Statistical analyses

Analytical data represented as histograms were recorded as mean values with bars representing standard errors of the mean (SEM) from a minimum of three independent experiments, with data obtained in triplicates, unless otherwise mentioned. Normality was assessed using D’Agostino & Pearson omnibus normality or Shapiro-Wilk (Royston) tests. All comparisons were performed using two-sided unpaired Student’s *t*-test, one-way analysis of variance (ANOVA) with a *post hoc* Bonferroni correction for multiple group comparisons or one-tailed z-test for comparing fractions. A *P-* value was assigned to each group with reference to control group, unless shown specifically on the graph. A *P* value of *<*0.05 was considered significant. All statistical analysis was performed with Graph Pad Prism software (Graph Pad 5.0).

## Supplementary Material

Supplemental Material
