# Supplementary material for: Cathelicidin-mediated lipopolysaccharide signaling via intracellular TLR4 in colonic epithelial cells evokes CXCL8 production
Source: Gut Microbes. 2020 Jul 13;12(1):1785802. doi: 10.1080/19490976.2020.1785802 (PMC7524372; doi:10.1080/19490976.2020.1785802)
Supplement: Supplemental Material [file KGMI_A_1785802_SM2247.zip › Supplementary information/Supporting information.docx]

**Supporting information**

**Supplementary Figure 1. *Citrobacter rodentium* killing by neutrophils isolated from *Camp*^+/+^** **and *Camp^-/-^* mice; determination of cecum weight, bacterial burden in liver and spleen and IL-1β, TNF-α and CXCL1 secretions in *Citrobacter rodentium* infected *Camp*^+/+^** **and *Camp^-/-^* mice; and cathelicidin expression in *Camp*^+/+^ mice. (A)** *In vitro* killing of *C. rodentium* by *Camp^+/+^*, *Camp^-/-^* or a 1:1 mix of *Camp^+/+^*and *Camp^-/-^* neutrophils. *C. rodentium* growth in LB media was used as reference to enumerate bacterial killing. **(B-C, E)** *Camp^+/+^* and *Camp^-/-^* mice were infected by *C. rodentium* (1 x 10^8^ CFU in 200 μL of PBS) for 7 d. **(B)** Representative pictures for appearance of cecum in both *Camp*^+/+^ and *Camp^-/-^* mice, and a bar graph representing total cecum weight. **(C)** A table depiciting fraction of mice positive for *C. rodentium* infiltration in liver and spleen of infected *Camp*^+/+^ and *Camp^-/-^* mice. **(D-E)** IL-1β and TNF-α secretions in distal colonic mucosa of *Camp^+/+^* and *Camp^-/-^* mice infected with *C. rodentium* (7 d pi; ELISA). **(F)** CXCL1 secretions in CD45^+^ CD326^-^ leukoctyes isolated from the lamina propria of colons of *Camp^+/+^* and *Camp^-/-^* mice infected with *C. rodentium* (7 d pi; ELISA). **(G)** *Camp* gene expression in CD326^+^ CD45^-^ colonic epithelium and lamina propria CD45^+^ CD326^-^ leukoctyes isolated from the colons of *Camp*^+/+^ mice (qPCR). **(A)** Statistical comparisions are made relative to “RPMI media” group. Data are shown as means ± SEM (n=4-6/group). *P* < 0.05 (One-way ANOVA *post hoc* Bonferroni correction for multiple group comparison, two-tailed Student’s *t-*test for two groups or z-test for comparing fractions) was considered significant.

**Supplementary Figure 2. CXCL8 secretion in presence of variable stimuli; MPO activity in colons of *Camp^-/-^* mice challenged by LPS and LL-37; *S. typhimurium* induced cytotoxicity in colonic epithelial cells (sham transfected or knocked down in LL-37) and; CXCL8 and TNF-α synthesis in PMA differentiated THP-1 monocytes in presence of LPS and LL-37. (A-B)** HT29 cells were challenged with LPS, flagellin and ODN (all 1 μg/mL, 16 h) **(A)** or heat inactivated *S. typhimurium* (2 x 10^8^ CFU equivalent; 70˚C for 15 min) for 4 h **(B)** either alone (except scrambled LL-37) and/or in combination with LL-37. **(A-B)** CXCL8 secretions were quantified as absolute values (pg/mL) using ELISA. **(C)** MPO activity was determined in homogenized colons of *Camp^-/-^* mice (*n*= 4/per group) treated with LPS (1 μg/g or 1 μg/mL), LL-37 (1 μg/g or 10 μg/mL), either alone or in combination (intraperitoneally for 3 h). **(D)** Cellular toxicity was evaluated by LDH assays in HT29 cells, either untransfected or transfected with sham plasmid/knocked-down for LL-37 (ShLL-37), infected with *S. typhimurium* (2 x 10^7^ CFU). Data were represented as percentage increase in LDH activity normalized to control. **(E)** PMA differentiated THP-1 monocytes were treated with LPS ± LL-37 for 4 h. Supernatants were evaluated for secretion of CXCL8 and TNF-α using ELISA kits. Data are shown as mean ± SEM (*n*= 3 independent experiments done in triplicate, unless mentioned otherwise in sub-figures). *P* < 0.05 (one-way ANOVA *post hoc* Bonferroni correction for multiple group comparison or two-tailed Student’s *t*-test for two groups) was considered significant. ND= “not detected”.

**Supplementary Figure 3. CXCL8 secretion in presence of variable stimuli, TLR4 expression and time dependent Alexa488-LPS uptake either alone or in combination with scrambled LL-37 (sLL-37) in colonic epithelial cells, and caspase-11 activation in murine colonoids. (A)** HT29 cells were pre-treated with the inhibitor LPS-RS (5 μg/mL) or L48H37 (10 μM) followed by LPS and LL-37, either alone or in combination. CXCL8 secretion was determined using ELISA after 4 h. **(B)** TLR4 expression in non-permeabilized (surface) and permeabilized (intracellular) HT29 cells as assessed by fluorescence microscopy using anti-TLR4 antibody (1:100; yellow) and a histogram of FACS based quantification using PE-labelled anti-TLR4-CD284 antibody (0.5 μg/mL). Isotype mouse IgG was used as a negative control in FACS. **(C)** HT29 cells were challenged with Alexa488-conjugated-LPS (1 μg/mL) ± sLL-37 (10 μg/mL) for variable interval (up to 4 h) and fluorescent images were obtained using wide field microscope. **(D)** HT29 cells were treated with LPS and CTB (10 μg/mL), either alone or in combination. CXCL8 secretion was determined using ELISA after 4 h. **(E)** *Camp^-/-^* murine colonoids were treated with LPS and LL-37, either alone or in combination (2 h) and blotted for cleaved caspase-11. GAPDH was used as house-keeping control. Data are shown as means ± SEM (*n*= 3 independent experiments done in triplicate, unless mentioned specifically in respective sub-figure). *P* < 0.05 (one-way ANOVA *post hoc* Bonferroni correction for multiple group comparison or two-tailed Student’s *t-*test for two groups) was considered significant.

**Supplementary Figure 4. Determination of CXCL8 secretion in colonic epithelial cells in presence of various inhibitors, cathelicidin and LPS dependent NF-κB (p65) activation in colonic epithelial cells, LPS dependent NF-κB (p65) activation in bone marrow derived macrophages from *Camp*^+/+^** **and *Camp^-/-^* mice, and EGFR and NF-κB (p65) phosphorylation in colons of *Citrobacter rodentium* infected *Camp*^+/+^** **and *Camp^-/-^* mice. (A-C)** HT29 cells were pre-treated with inhibitors TAK165 (ErbB2; 10 μM), WRW4 (FPRL1; 10 μM), GM6001 (MMP; 10 μM) and A740003 (P2X7; 10 μM) **(A)**, not pre-treated **(B)** or pre-treated with MEK1/2 inhibitor (PD98059; 20 μM) **(C)**, followed by LPS and LL-37, either alone **(A and C)** or in combination **(B)**. **(D)** BMMs from *Camp^+/+^* and *Camp^-/-^* mice were challenged with LPS (1 μg/mL) for 2 h. **(A and C)** CXCL8 was quantified in cell supernatants using ELISA. **(B and D)** Cell lysate were blotted against phosphorylated p65 subunit of NF-κB. **(E)** Western blotting for phosphorylated EGFR and p65 subunit of NF-κB in distal colons of *C. rodentium* infected *Camp*^+/+^ and *Camp^-/-^* mice. GAPDH was used as housekeeping control. Data are shown as means ± SEM (*n*= 3 independent experiments done in triplicate, unless mentioned specifically in respective figure). *P* < 0.05 (one-way ANOVA *post hoc* Bonferroni correction for multiple group comparison or two-tailed Student’s *t-*test for two groups) was considered significant.

**Supplementary Figure 5. Dose and time curves for CXCL8 synthesis in colonic epithelial cells in presence of LPS and/or LL-37. (A-E)** HT29 cells were challenged with variable concentrations of LPS alone **(A)**, LL-37 alone **(B)**, constant LPS (1 μg/mL) and variable concentrations of LL-37 **(C)**, constant LL-37 (10 μg/mL) and variable concentrations of LPS **(D)**, or with LPS (1 μg/mL) and LL-37 (10 μg/mL) **(E)**. **(A-D)** CXCL8 secretions were assessed by ELISA after 4 h. **(E)** *CXCL8* mRNA synthesis was quantified using qPCR over a period of 4 h (top graph), and protein secretions were determined in cell supernatant for up to 16 h (bottom graph). Data are shown as means ± SEM (*n*= 3 independent experiments done in triplicate). *P*< 0.05 (One-way ANOVA *post hoc* Bonferroni correction) was considered significant.
