# Supplementary figures and images for: Cathelicidin-mediated lipopolysaccharide signaling via intracellular TLR4 in colonic epithelial cells evokes CXCL8 production
Source: Gut Microbes. 2020 Jul 13;12(1):1785802. doi: 10.1080/19490976.2020.1785802 (PMC7524372; doi:10.1080/19490976.2020.1785802)

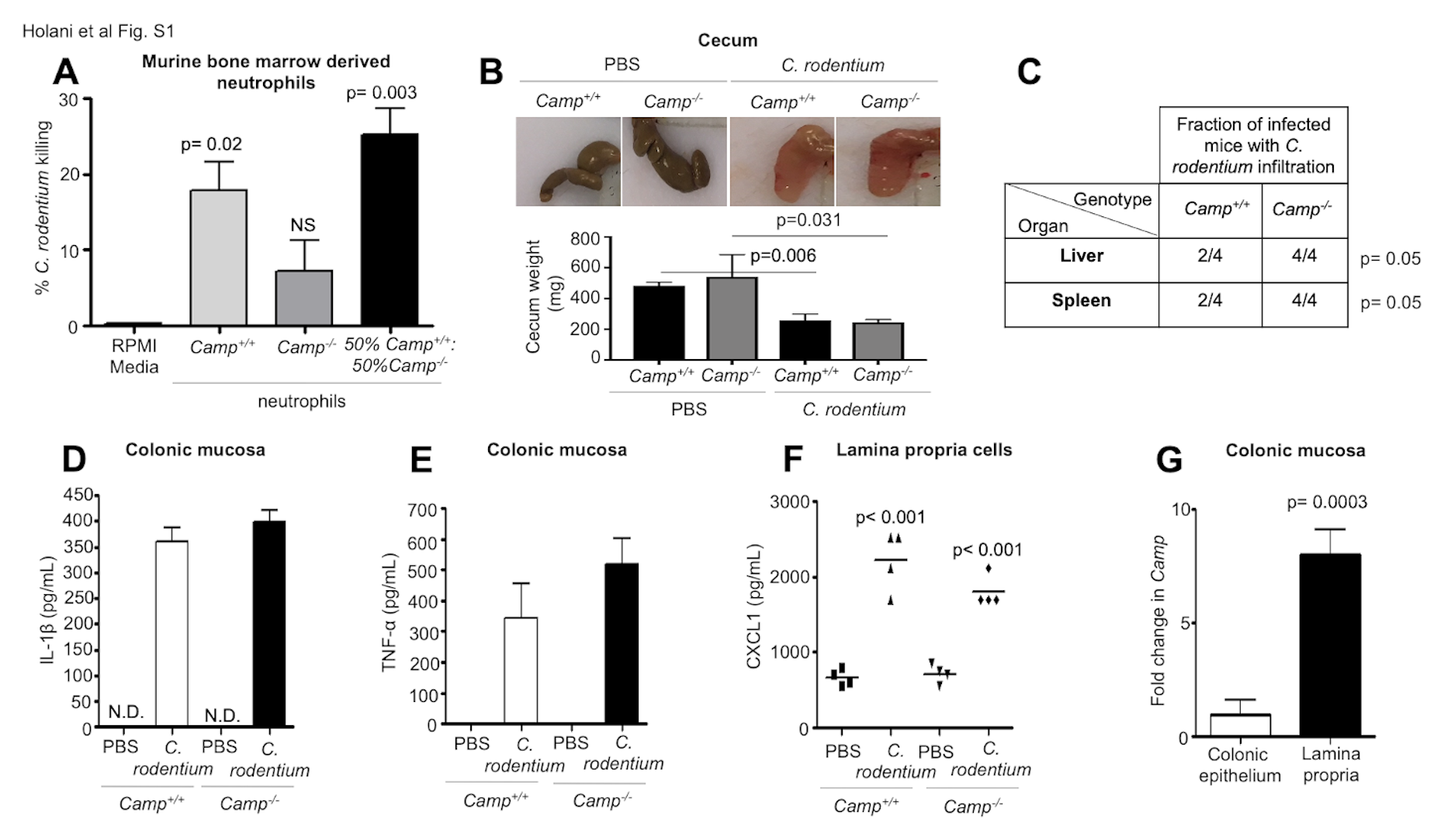

Supplement: Supplemental Material [file KGMI_A_1785802_SM2247.zip › Supplementary information/Holani et al (R2) Supp Figure 1.tif]

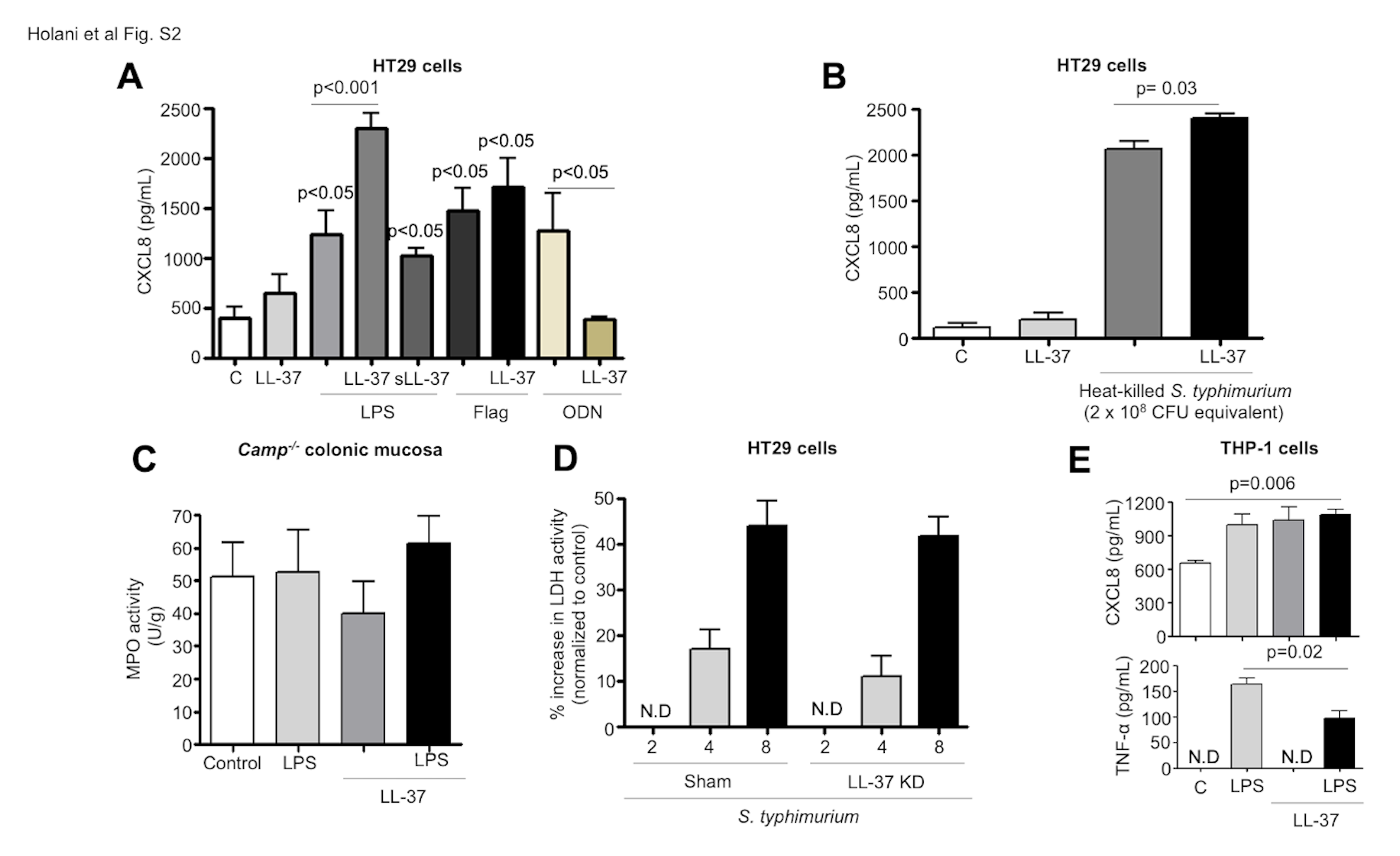

Supplement: Supplemental Material [file KGMI_A_1785802_SM2247.zip › Supplementary information/Holani et al (R2) Supp Figure 2.tif]

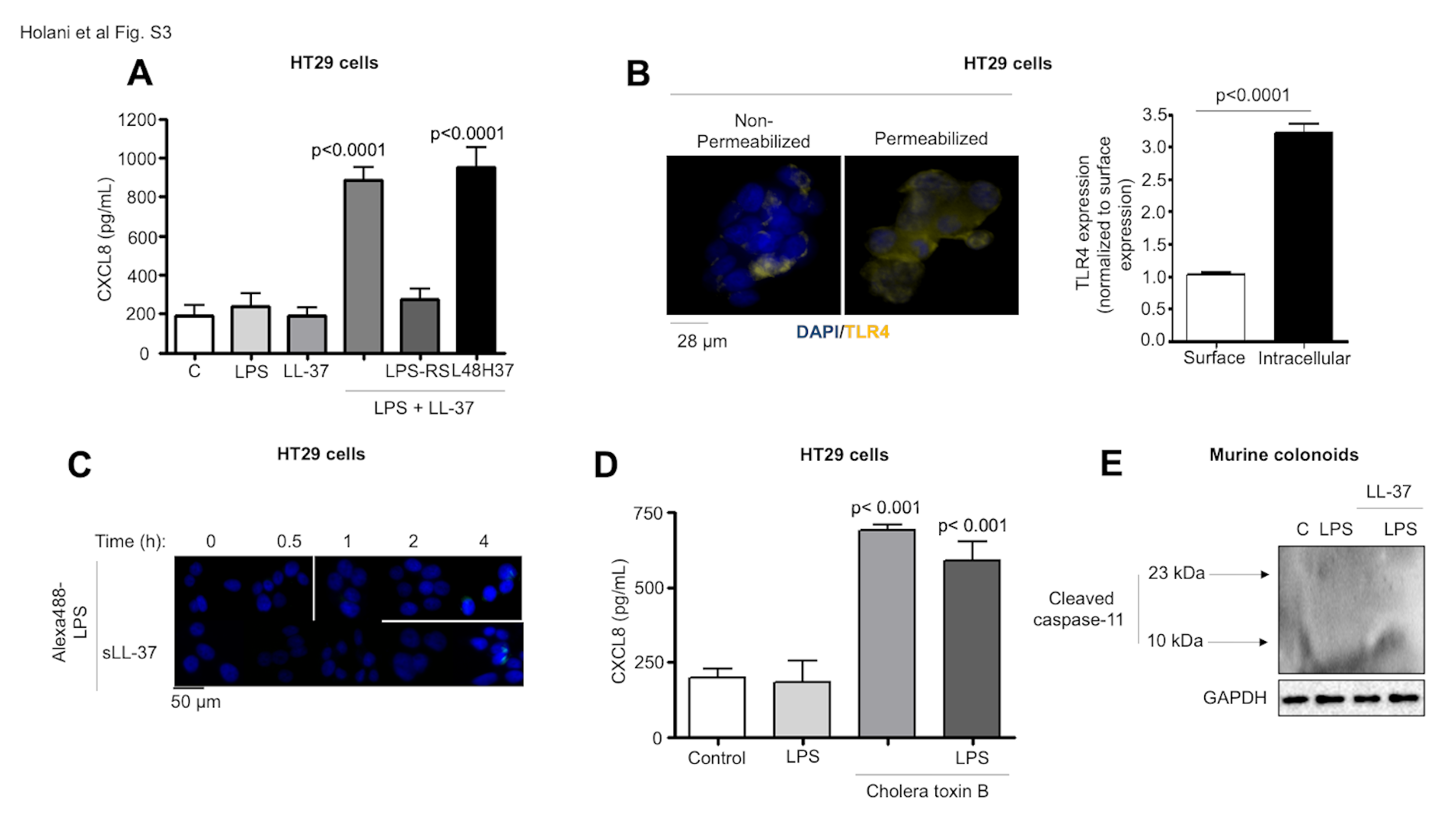

Supplement: Supplemental Material [file KGMI_A_1785802_SM2247.zip › Supplementary information/Holani et al (R2) Supp Figure 3.tif]

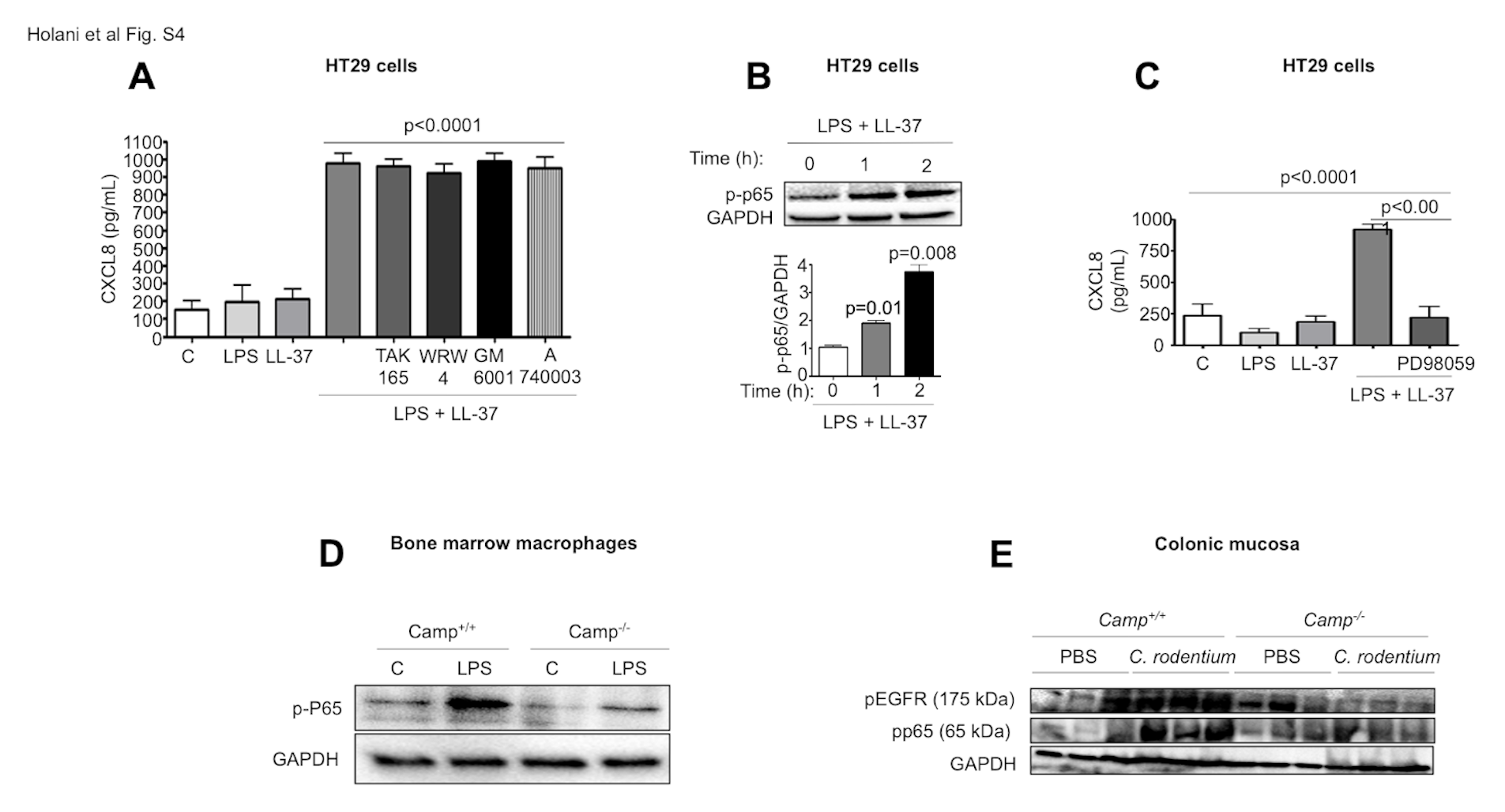

Supplement: Supplemental Material [file KGMI_A_1785802_SM2247.zip › Supplementary information/Holani et al (R2) Supp Figure 4.tif]

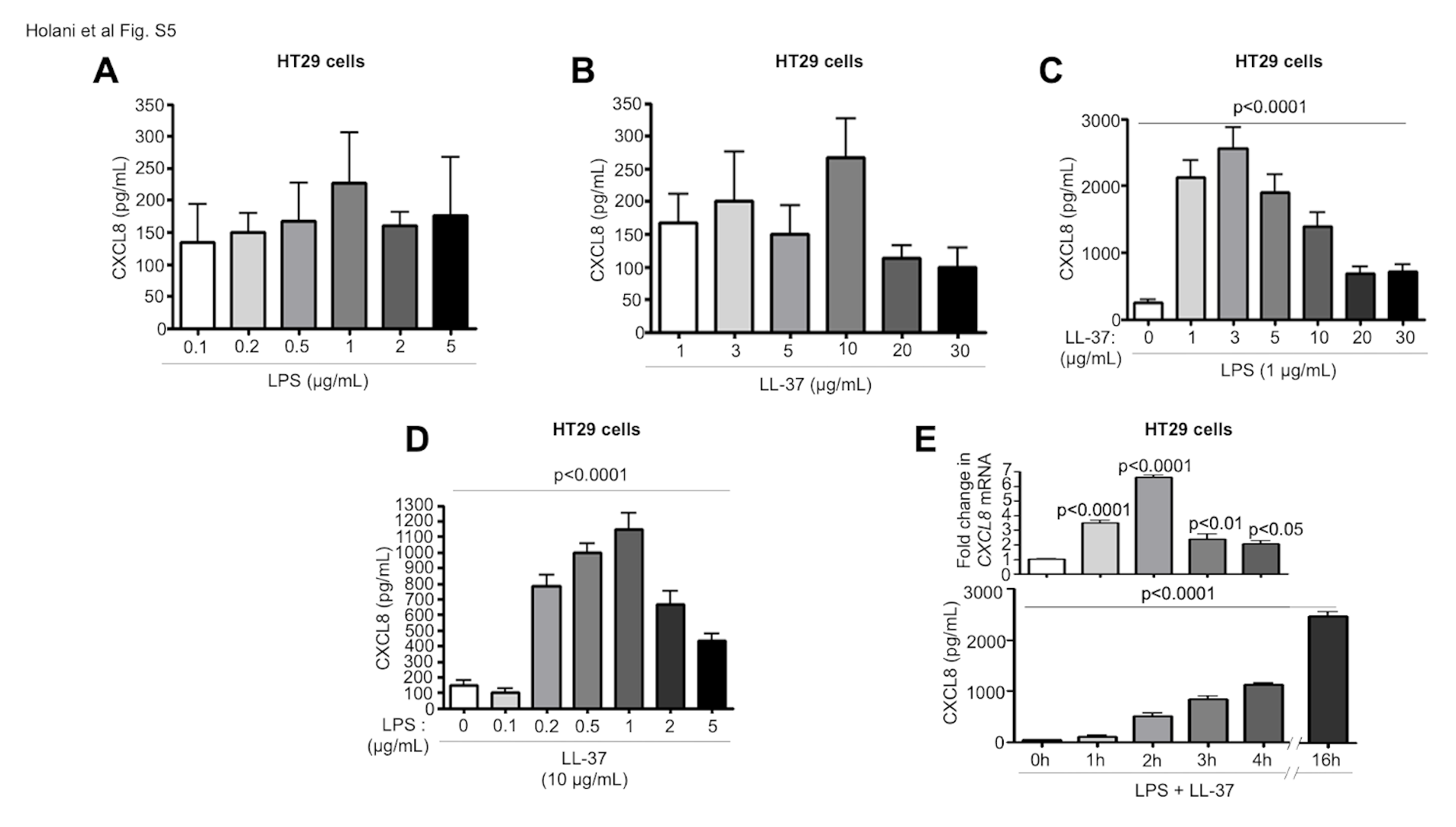

Supplement: Supplemental Material [file KGMI_A_1785802_SM2247.zip › Supplementary information/Holani et al (R2) Supp Figure 5.tif]
